# Research on panic spread and decision behaviour in a delayed SEIR evolutionary game model under an emergency

**DOI:** 10.1038/s41598-023-44116-4

**Published:** 2023-10-13

**Authors:** Rongjian Lv, Hua Li, Qiubai Sun, Bowen Li

**Affiliations:** 1 School of Electronics and Information Engineering, University of Science and Technology Liaoning, Anshan, Liaoning China; 2 School of Business Administration, University of Science and Technology Liaoning, Anshan, Liaoning China

**Keywords:** Computational biology and bioinformatics, Engineering, Mathematics and computing

## Abstract

Taking major emergencies as the research background, the transmission mechanism for panic spread and the decision behaviour of the opinion field are explored in this paper. By using evolutionary game theory to explore the game relationship between the official opinion field and the public opinion field and by considering the existence of strategy dependency in the same game group, the dependence coefficient is introduced to improve replicator dynamics. The dynamic delayed SEIR evolutionary game model is built by combining the epidemic model with the delay effect within the group, and the stability of the proposed model is analysed. The research results show that the strategy dependency among the same game group has positive and negative effects on the evolution process. The evolution of the dynamic delayed panic SEIR evolutionary game spread model under the effect of a positive effect is simulated. The results suggest that the official opinion field and the public opinion field should actively deal with emergencies, formulate effective control strategies to make the panic within the group disappear, and provide theoretical guidance for the relevant government to formulate plans.

## Introduction

In recent years, emergencies, such as earthquakes^1, 2^, public health events^3, 4^, and stampedes^5, 6^, have occurred frequently, seriously endangering the security and stability of society, generating serious human casualties and economic losses, and bringing negative impacts to society^7–9^.

Emergencies generally lead to the spread of false information and negative emotions within groups, and the government usually takes decisive measures to control them. Therefore, it is necessary to explore the mechanisms of the spread of information and emotion as well as that of decision-making behaviour. In general, epidemic models are widely used in the construction of spread models because of their characteristics^10^. The occurrence of emergencies causes false information to spread within groups, i.e., the spread of rumours^11, 12^. To determine the spread law of rumours within groups, Hu et al.^13, 14^ built a rumour spread model based on the epidemic model, considering the proportion of each type of group in the community and the individuals’ different attitudes towards rumour spread. The threshold and equilibrium point of the model are calculated to simulate the process of rumour spread. Tian^15^ developed an ILRDS rumour spread model, used the Poincarè–Bendixson theorem and a Lyapunov function to analyse the stability of the model, and simulated the dynamical behaviour of the model. To reduce the impact of rumour spreading within a group, Yi et al.^16^ introduced a rumour clarification node and proposed the twin-SIR spreading model to control the rumor spread and thus to clarify rumours. Based on the characteristics of rumour spread, Chen et al.^17^ considered the delay of interactive systems to build an improved rumour spread model. The conditions of rumour extinction in the model are calculated, the dynamic behaviour of the model is analysed, and relevant strategies are proposed to control rumour spreading. Hu^18^ proposed a reaction–diffusion rumour spread model with a time delay based on changes in complex network models. The diffusion and Turing pattern near the equilibrium point of the model are investigated and fitted to real data, and the results verify that the proposed model is effective. The spread of false information within a group leads to the spread of negative emotions such as panic within the group^19, 20^. Xu et al.^21^ developed a group movement model to consider the effects of initial negative pedestrian proportion, pedestrian crowd density and emotion influence radius influence on the group's emotional state transition. Group movement can be influenced by individual emotions. Under emergencies, individuals are also be affected by the emotions of others, which makes it easier for people to gather^22^. Li^23^ considered the delay effect of susceptible individuals transforming into infected individuals, and the delayed panic spread model was established. The effects of delay, infection rate, and initial density of the three categories of people on the panic spread were analysed to provide theoretical guidance for the development of emergency plans by the relevant departments.

Emergencies can quickly escalate in a short period. If the government fails to take control and to take decision-making actions, the result can be more serious consequences than those from the outbreak itself. Xia et al.^24^ constructed the SIR rumour spreading model with general nonlinear functional reaction–diffusion and proved the global existence and limit boundedness of the model solution. An optimal strategy combining an educational mechanism and an enforced silence method was proposed to control rumour spreading. Chen et al.^25^ proposed a heterogeneous complex network delayed SEIR rumour spread model with saturation incidence, and they calculated the basic regeneration number and equilibrium point of the model. The optimal strategy of the optimal control model was analysed by Pontryagin's principle. Cheng^26^ established an optimal control model of delayed rumour spread with pulse vaccination, and the results showed that pulse measures can control rumour spread and can reduce the time of rumour spread to minimize damage. Wang et al.^27^ proposed an improved computational model for the SLIRS spread of panic, based on which the optimal control problem was established. Simulated data and real datasets were employed for numerical simulations, and the results show that the optimal control strategy was effective in suppressing emotional contagion. The research suggests that decision-making behaviour after emergencies can be explored by using evolutionary game theory^28, 29^. Li et al.^27^ and Li^30, 31^ applied evolutionary game theory, and an evolutionary game model was constructed to analyse the behavioural evolution process of the various groups affected by disinformation in major emergencies. Wang^32^ established an evolutionary game theory model to analyse the behavioural evolution process of netizens who are affected by negative information during emergencies. Shi et al.^33^ used a three-way evolutionary game model to analyse the dynamic evolutionary process of evacuation decisions of different decision-makers during emergencies. Kabir ^34^ developed an evolutionary game model for socioeconomic costs in emergencies, and the results showed that controlling boundary measurements work well in the final stage of the emergency with lower costs. Wang et al.^35^ considered that individuals act rationally rather than panic under emergencies, and the evolutionary behaviour of large-scale populations was studied based on evolutionary game theory and evolutionary rules.

In summary, the epidemic model is an effective tool to study the law of spread, while optimal control theory and game theory can be used to analyse the decision behaviour of opinion fields in response to emergencies. At present, scholars usually analyse the influence of various opinion fields on the spread of panic within a group as a fixed parameter, while in actuality, there exists a game relationship between each side of the opinion field (the official opinion field and the public opinion field), which has a different influence on the panic spread under emergencies and is defined as a time-varying variable. Moreover, the dependency coefficients are used to indicate the dependency between the strategies of the same game group and to improve the traditional replicated dynamic equations. Therefore, in this paper, the evolutionary game model and the epidemic model are combined, while considering the delay effect in the epidemic model; the dynamic delayed SEIR evolutionary game model was established to analyse the study of the propagation and control of panic under emergencies. The rest of this article is structured as follows. In Sect. "Model formulation", the dynamic delayed panic SEIR evolutionary game model is developed. The stability of the proposed model is analysed in Sect. "Stability analysis". In Sect. "Numerical simulation", numerical simulations are performed to verify the theoretical results. A short conclusion is given in Section "Conclusion".

## Model formulation

### Improved evolutionary game model

Considering the existence of dependency between the strategies adopted by the same game, the dependence coefficient is introduced to enrich evolutionary game theory^36^. There are *n* state spaces in game Group *D*. The number of individuals who select strategy *i* at time *t* is $n_{i} (t)$, the adaptive capacity is $u_{i}^{D} (t)$, the probability of accounting for Group *D* is $z_{i} (t)$, and the average adaptive capacity of game Group *D* at time *t* is $\overline{u}^{D} (t)$. We find that

1 $$\sum\limits_{i = 1}^{n} {z_{i} (t)} = 1$$

2 $$z_{i} (t) = \frac{{n_{i} (t)}}{{\sum\limits_{i = 1}^{m} {n_{i} (t)} }}$$

3 $$\overline{u}^{D} (t) = \sum\limits_{i = 1}^{m} {z_{i} (t)} u_{i}^{D} (t)$$

The number of individuals who select strategy *i* changes over time and is positively correlated with the number of individuals who select strategy *i* at time *t* and with adaptive capacity, i.e.,

4 $$n_{i}^{\prime } (t) = \kappa_{i} n_{i} (t)u_{i}^{D} (t)$$

where $\kappa_{i}$ is the strategy influence coefficient, which indicates the strength of influence of strategy *i* on other strategies within the game group. Differentiation of (2) yields the following improved replication dynamic:

5 $$\begin{gathered} z^{\prime}_{i} (t) = \frac{{n^{\prime}_{i} (t)\sum\limits_{i = 1}^{m} {n_{i} (t)} - n_{i} (t)\sum\limits_{i = 1}^{m} {n^{\prime}_{i} (t)} }}{{\left[ {\sum\limits_{i = 1}^{n} {n_{i} (t)} } \right]^{2} }} \hfill \\ {\kern 1pt} {\kern 1pt} {\kern 1pt} {\kern 1pt} {\kern 1pt} {\kern 1pt} {\kern 1pt} {\kern 1pt} {\kern 1pt} {\kern 1pt} {\kern 1pt} {\kern 1pt} {\kern 1pt} {\kern 1pt} {\kern 1pt} {\kern 1pt} {\kern 1pt} {\kern 1pt} {\kern 1pt} {\kern 1pt} {\kern 1pt} {\kern 1pt} {\kern 1pt} {\kern 1pt} { = }z_{i} (t)\kappa_{i} \left[ {u_{i}^{D} (t) - \overline{u}^{D} (t) + \sum\limits_{\alpha = 1}^{m} {(1 - \frac{{\kappa_{\alpha } }}{{\kappa_{i} }})z_{\alpha } (t)u_{\alpha }^{D} (t)} } \right] \hfill \\ \end{gathered}$$

where $\frac{{\kappa_{\alpha } }}{{\kappa_{i} }}$ is the dependence coefficient between the strategies of the same game.

### Improved evolutionary game

In the process of spreading panic under emergencies, there is a game relationship between the official opinion field and the public opinion field. The former is structured by National Television and National News Agency such as TV, radio and newspapers. The aim is to maintain the overall situation, speaking cautiously and establish official credibility. In contrast, the latter is formed by teach orally, in recent years, by using network and other modes to express public opinion from their own interests. Therefore, to construct an improved evolutionary game model between the official public opinion field and the private public opinion field under emergencies, the assumptions are as follows:

1. The set of strategies in the official opinion field is positive guidance and negative guidance. The probability of the official opinion field choosing positive guidance is $x_{1}$, so the probability of the official opinion field choosing negative guidance is $x_{2}$ ($x_{2} = 1 - x_{1}$) . The set of strategies in the public opinion field includes positive and negative responses. The probability of the public opinion field releasing a positive response is $y_{1}$, so the probability of the public opinion field releasing a negative response is $y_{2}$ ($y_{2} { = }1 - y_{1}$) .
2. When the attitude of the public opinion field to take measure is positive, whether positive guidance or negative guidance, the benefit of the official opinion field is $B_{1}$. Moreover, when the attitude of the public opinion field to take measure is negative, whether positive guidance or negative guidance, the benefit of the official opinion field is $B_{2}$. In the face of emergencies, the official opinion field is required to pay the fixed cost of $C_{1}$. When the public opinion field adopts positive response, in order to cooperate with the decision-making of the public opinion field, the official opinion field will pay the additional cost as $C_{11}$, whether it adopts positive guidance or negative guidance.
3. When the public opinion field adopts a positive response and the official public opinion adopts a positive guidance, the benefit is $B_{3}$. When the official opinion field adopts positive guidance, in order to cooperate with the decision-making of the official opinion field, the public opinion field will pay the additional cost as $C_{21}$, whether it adopts positive response or negative response. When the public opinion field adopts a negative response and the official public opinion adopts a positive guidance, the benefit is $B_{4}$. When the public opinion field adopts a positive response and the official opinion field adopts negative guidance, the benefit is $B_{5}$. When the public opinion field adopts a negative response and the official opinion field adopts negative guidance, the benefit is $B_{6}$.
4. All the parameters mentioned above are greater than zero, and $0 \le x_{1} \le 1$,$0 \le y_{1} \le 1$,$B_{1} > B_{2}$,$B_{3} > B_{4} > B_{5} > B_{6}$.

Based on assumptions (1)–(4), the income of each game participant for each combination of strategies is shown in Table 1.

**Table 1:** The two parties game income matrix for the official opinion field and the public opinion field.

		Public opinion field	
		Positive response	Negative response
Official opinion field	Positive guidance	*B*_1_-*C*_1_-*C*_11_ + *C*_21_, *B*_3_-*C*_21_	*B*_2_-*C*_1_ + *C*_21_, *B*_4_-*C*_21_
	Negative guidance	*B*_1_-*C*_1_-*C*_11_, *B*_5_	*B*_2_-*C*_1_, *B*_6_

Based on the income matrix shown in Table 1, $u_{1}^{A}$ and $u_{2}^{A}$ are writing for the income when the official opinion field takes positive guidance and negative guidance, respectively. $\overline{u}^{A}$ denotes the average income of the official opinion field. The formulas are shown below:

6 $$u_{1}^{A} = y_{1} (B_{1} - C_{1} - C_{11} + C_{21} ) + y_{2} (B_{2} - C_{1} + C_{21} )$$

7 $$u_{2}^{A} = y_{1} (B_{1} - C_{1} - C_{11} ) + y_{2} (B_{2} - C_{1} )$$

8 $$\overline{u}^{A} = x_{1} u_{1}^{A} + x_{2} u_{2}^{A}$$

Similarly, $u_{1}^{B}$ and $u_{2}^{B}$ are used for the income when the public opinion field takes a positive response and a negative response, respectively. $\overline{u}^{B}$ denotes the average income of the public opinion field. The formulas are shown below:

9 $$u_{1}^{B} = x_{1} (B_{3} - C_{21} ) + x_{2} B_{5}$$

10 $$u_{2}^{B} = x_{1} (B_{4} - C_{21} ) + x_{2} B_{6}$$

11 $$\overline{u}^{B} (t) = y_{1} u_{1}^{B} + y_{2} u_{2}^{B}$$

Since $x_{1} + x_{2} = 1$, $y_{1} + y_{2} = 1$.$x^{\prime}_{1} = - x^{\prime}_{2}$, $y^{\prime}_{1} = - y^{\prime}_{2}$ can be obtained. Because of the dependence of the respective strategies of the same game group, the official opinion field and the public opinion field, the improved replicated dynamic is used to describe the evolutionary game model^36^. Combined with Eqs. (5)– (11), the improved replicated dynamic of the official opinion field and the public opinion field are obtained:

12 $$\begin{aligned} \frac{{dx_{1} (t)}}{{dt}} = & \lambda _{1} x_{1} x_{2} \{ (B_{2} - C_{1} + C_{{21}} ) - p_{{21}} (B_{2} - C_{1} ){\kern 1pt} \\ {\kern 1pt} & + [(B_{1} - C_{1} - C_{{11}} + C_{{21}} ) - (B_{2} - C_{1} + C_{{21}} ) - p_{{21}} (B_{1} - C_{1} - C_{{11}} ) + p_{{21}} (B_{2} - C_{1} )]y_{1} \} \\ {\kern 1pt} & = \lambda _{1} x_{1} x_{2} \{ (B_{2} - C_{1} + C_{{21}} ) - p_{{21}} (B_{2} - C_{1} ) + [(1 - p_{{21}} )(B_{1} - C_{{11}} - B_{2} )]y_{1} \} \\ {\kern 1pt} & = \lambda _{1} x_{1} (1 - x_{1} )\{ (B_{2} - C_{1} + C_{{21}} ) - p_{{21}} (B_{2} - C_{1} ) + [(1 - p_{{21}} )(B_{1} - C_{{11}} - B_{2} )]y_{1} \} \\ \end{aligned}$$

13 $$\begin{aligned} \frac{{dy_{1} (t)}}{{dt}} = & \rho _{1} y_{1} y_{2} \{ B_{5} - q_{{21}} B_{6} + [(B_{3} - C_{{21}} ) - B_{5} - q_{{21}} (B_{4} - C_{{21}} ) + q_{{21}} B_{6} ]x_{1} \} \\ {\kern 1pt} & {\kern 1pt} = \rho _{1} y_{1} (1 - y_{1} )\{ B_{5} - q_{{21}} B_{6} + [B_{3} - C_{{21}} - B_{5} - q_{{21}} (B_{4} - C_{{21}} ) + q_{{21}} B_{6} ]x_{1} \} \\ \end{aligned}$$

where $p_{21} = \frac{{\lambda_{2} }}{{\lambda_{1} }}$ and $q_{21} = \frac{{\rho_{2} }}{{\rho_{1} }}$ are the dependence coefficients of the same game group, which are the official opinion field and the public opinion field, respectively ^36^.

### Delayed panic SEIR spread model

In an emergency, panic can spread within the group. Motivated by Refs. ^37^, the group can be divided into four compartments, namely, $S(t),E(t),I(t),R(t)$. Susceptible (people who are vulnerable to panic), exposed (people who have only symptoms of infection without the ability to spread panic), infective (people who spread panic) and recovered (people who have experienced panic but never spread panic) individuals.

The panic within the group is influenced by the official opinion field. Because of its authority *m*, susceptible individuals are transformed into recovered individuals with a probability of $mx_{1}$. In contrast, due to the negative guidance of the official opinion field, the exposed individuals are transformed into infected individuals with a probability of $mx_{2}$. As a result of the positive response in the public opinion field, the exposed individuals are transformed into recovered individuals with a probability of $y_{1}$. Conversely, due to the negative response in the public opinion field, exposed individuals are transformed into the infected individuals with a probability of $y_{2}$. The infected individuals are transformed into recovered individuals with recovery rate $\gamma$. The recovered individuals experience permanent immunity with probability, i.e., they cannot become susceptible individuals again.

The Holling-type II functional response function was used to represent the infection rate^38^:

14 $$\phi (I)S = \frac{\beta SI}{{1 + \alpha I}}$$

where $\phi (I)$ indicates the number of groups per unit of time and represents the functional response function.

In contrast, there is a delay effect between the transformation of susceptible individuals to exposed individuals in the emergency. Based on this, the dynamic delayed SEIR evolutionary game model is constructed. The model can be described as

15 $$\left\{ \begin{gathered} \frac{dS}{{dt}} = a - \frac{\beta SI(t - \tau )}{{1 + \alpha I(t - \tau )}} - mx_{1} S \hfill \\ \frac{dE}{{dt}} = \frac{\beta SI(t - \tau )}{{1 + \alpha I(t - \tau )}} - mx_{2} E - y_{1} E + y_{2} I \hfill \\ \frac{dI}{{dt}} = mx_{2} E - \gamma I - y_{2} I \hfill \\ \frac{dR}{{dt}} = \gamma I + mx_{1} S + y_{1} E \hfill \\ \end{gathered} \right.$$

## Stability analysis

### Stability analysis of the improved evolutionary game model

The values of (12) and (13) are set to 0. The five stable equilibrium points of the evolutionary game model are obtained as $E_{1} (0,0)$, $E_{2} (0,1)$, $E_{3} (1,0)$, $E_{4} (1,1)$ and $E_{5} (\frac{{B_{5} - q_{21} B_{6} }}{{B_{5} - B_{3} + C_{21} + q_{21} (B_{4} - C_{21} ) - q_{21} B_{6} }},\frac{{B_{2} - C_{1} + C_{21} - p_{21} (B_{2} - C_{1} )}}{{(1 - p_{21} )(B_{2} + C_{11} - B_{1} )}})$, respectively.

The system dynamics approach^39, 40^ was used to analyse the improved replication dynamic equations in the evolutionary game model. The Jacobian matrix of the evolutionary game model is as follows:

16 $$J = \left[ {\begin{array}{*{20}c} {\frac{{\partial F_{1} (x_{1} ,y_{1} )}}{{\partial x_{1} }}} & {\frac{{\partial F_{1} (x_{1} ,y_{1} )}}{{\partial y_{1} }}} \\ {\frac{{\partial F_{2} (x_{1} ,y_{1} )}}{{\partial x_{1} }}} & {\frac{{\partial F_{2} (x_{1} ,y_{1} )}}{{\partial y_{1} }}} \\ \end{array} } \right]$$

where

$$\frac{{\partial F_{1} (x_{1} ,y_{1} )}}{{\partial x_{1} }} = \lambda_{1} (1 - 2x_{1} )\{ (B_{2} - C_{1} + C_{21} ) - p_{21} (B_{2} - C_{1} ) + [(1 - p_{21} )(B_{1} - C_{11} - B_{2} )]y_{1} \}$$

$$\frac{{\partial F_{1} (x_{1} ,y_{1} )}}{{\partial y_{1} }} = \lambda_{1} x_{1} (1 - x_{1} )(1 - p_{21} )(B_{1} - C_{11} - B_{2} )$$

$$\frac{{\partial F_{2} (x_{1} ,y_{1} )}}{{\partial x_{1} }} = \rho_{1} y_{1} (1 - y_{1} )[B_{3} - C_{21} - B_{5} - q_{21} (B_{4} - C_{21} ) + q_{21} B_{6} ]$$

$$\frac{{\partial F_{2} (x_{1} ,y_{1} )}}{{\partial y_{1} }} = \rho _{1} (1 - 2y_{1} )\{ B_{5} - q_{{21}} B_{6} + [B_{3} - C_{{21}} - B_{5} - q_{{21}} (B_{4} - C_{{21}} ) + q_{{21}} B_{6} ]x_{1} \}$$

From the Jacobian (16), the determinant and the trace of the matrix are obtained:

17 $$\begin{aligned} \det J = & \lambda _{1} (1 - 2x_{1} )\{ (B_{2} - C_{1} + C_{{21}} ) - p_{{21}} (B_{2} - C_{1} ) + [(1 - p_{{21}} )(B_{1} - C_{{11}} - B_{2} )]y_{1} \} \cdot \\ {\kern 1pt} {\kern 1pt} & \rho _{1} (1 - 2y_{1} )\{ B_{5} - q_{{21}} B_{6} + [B_{3} - C_{{21}} - B_{5} - q_{{21}} (B_{4} - C_{{21}} ) + q_{{21}} B_{6} ]x_{1} \} - \\ {\kern 1pt} & \lambda _{1} x_{1} (1 - x_{1} )(1 - p_{{21}} )(B_{1} - C_{{11}} - B_{2} ) \cdot \\ {\kern 1pt} {\kern 1pt} & {\kern 1pt} \rho _{1} y_{1} (1 - y_{1} )[B_{3} - C_{{21}} - B_{5} - q_{{21}} (B_{4} - C_{{21}} ) + q_{{21}} B_{6} ] \\ \end{aligned}$$

18 $$\begin{aligned} trJ = & \lambda _{1} (1 - 2x_{1} )\{ (B_{2} - C_{1} + C_{{21}} ) - p_{{21}} (B_{2} - C_{1} ) + [(1 - p_{{21}} )(B_{1} - C_{{11}} - B_{2} )]y_{1} \} + \\ {\kern 1pt} & \rho _{1} (1 - 2y_{1} )\{ B_{5} - q_{{21}} B_{6} + [B_{3} - C_{{21}} - B_{5} - q_{{21}} (B_{4} - C_{{21}} ) + q_{{21}} B_{6} ]x_{1} \} \\ \end{aligned}$$

Since the parameter value of the improved evolutionary game is undetermined, the symbol of the determinant and trace cannot be determined, which makes it difficult to judge the stability of the improved evolutionary game model. Therefore, it needs to be discussed in four cases, in the form shown below:

(I) When $B_{5} - B_{3} + C_{21} + q_{21} (B_{4} - C_{21} ) - q_{21} B_{6} = 0$ and $(1 - p_{21} )(B_{2} + C_{11} - B_{1} ) = 0$, the four equilibrium points of the evolutionary game model are $E_{1} (0,0)$, $E_{2} (0,1)$, $E_{3} (1,0)$ and $E_{4} (1,1)$, respectively. Substituting the equilibrium points into Eqs. (17) and (18), we can obtain the determinant and trace of the Jacobian matrix of the improving replicator dynamic. The specific results are shown in Table 2.

**Table 2:** The determinant and trace of the Jacobian matrix corresponding to the equilibrium point of the improving replicator dynamic.

Equilibrium points	The determinant and trace of the Jacobian matrix
$E_{1} (0,0)$	$\det J = \lambda_{1} \rho_{1} [(B_{2} - C_{1} + C_{21} ) - p_{21} (B_{2} - C_{1} )](B_{5} - q_{21} B_{6} )$
	$$trJ = \lambda_{1} [(B_{2} - C_{1} + C_{21} ) - p_{21} (B_{2} - C_{1} )] + {\kern 1pt} \rho_{1} (B_{5} - q_{21} B_{6} )$$
$E_{2} (0,1)$	$\det J = - \lambda_{1} \rho_{1} (B_{5} - q_{21} B_{6} ){[(}B_{1} - C_{1} - C_{11} + C_{21} {)} - p_{21} {(2}B_{2} - B_{1} - C_{1} + C_{11} {)]}$
	$$trJ = \lambda_{1} {[(}B_{1} - C_{1} - C_{11} + C_{21} {)} - p_{21} {(2}B_{2} - B_{1} - C_{1} + C_{11} {)]} - \rho_{1} (B_{5} - q_{21} B_{6} )$$
$E_{3} (1,0)$	$\det J = - \lambda_{1} \rho_{1} [(B_{2} - C_{1} + C_{21} ) - p_{21} (B_{2} - C_{1} )][(B_{3} - C_{21} ) - q_{21} (B_{4} - C_{21} )]$
	$$trJ = - \lambda_{1} [(B_{2} - C_{1} + C_{21} ) - p_{21} (B_{2} - C_{1} )] + \rho_{1} [(B_{3} - C_{21} ) - q_{21} (B_{4} - C_{21} )]$$
$E_{4} (1,1)$	$$\begin{gathered} \det J = \lambda_{1} \rho_{1} {[(}B_{1} - C_{1} - C_{11} + C_{21} {)} - p_{21} {(2}B_{2} - B_{1} - C_{1} + C_{11} {)]} \cdot \hfill \\ {\kern 1pt} {\kern 1pt} {\kern 1pt} {\kern 1pt} {\kern 1pt} {\kern 1pt} {\kern 1pt} {\kern 1pt} {\kern 1pt} {\kern 1pt} {\kern 1pt} {\kern 1pt} {\kern 1pt} {\kern 1pt} {\kern 1pt} {\kern 1pt} {\kern 1pt} {\kern 1pt} {\kern 1pt} {\kern 1pt} {\kern 1pt} {\kern 1pt} {\kern 1pt} {\kern 1pt} {\kern 1pt} {\kern 1pt} {\kern 1pt} {\kern 1pt} {\kern 1pt} {\kern 1pt} {\kern 1pt} {\kern 1pt} {\kern 1pt} {\kern 1pt} {\kern 1pt} {\kern 1pt} {\kern 1pt} {\kern 1pt} [(B_{3} - C_{21} ) - q_{21} (B_{4} - C_{21} )] \hfill \\ \end{gathered}$$
	$$\begin{gathered} trJ = - \lambda_{1} {[(}B_{1} - C_{1} - C_{11} + C_{21} {)} - p_{21} {(2}B_{2} - B_{1} - C_{1} + C_{11} {)]} \hfill \\ {\kern 1pt} {\kern 1pt} {\kern 1pt} {\kern 1pt} {\kern 1pt} {\kern 1pt} {\kern 1pt} {\kern 1pt} {\kern 1pt} {\kern 1pt} {\kern 1pt} {\kern 1pt} {\kern 1pt} {\kern 1pt} {\kern 1pt} {\kern 1pt} {\kern 1pt} {\kern 1pt} {\kern 1pt} {\kern 1pt} {\kern 1pt} {\kern 1pt} {\kern 1pt} {\kern 1pt} {\kern 1pt} - \rho_{1} [(B_{3} - C_{21} ) - q_{21} (B_{4} - C_{21} )] \hfill \\ \end{gathered}$$

The determinant and trace of the Jacobian matrix are discussed for each case:

1. When ${(}B_{1} - C_{1} - C_{11} + C_{21} {)} - p_{21} {(}B_{1} - C_{1} - C_{11} {)} < 0$,$(B_{3} - C_{21} ) - q_{21} (B_{4} - C_{21} ) < 0$, $E_{1} (0,0)$ is the stable point, $E_{2} (0,1)$ and $E_{3} (1,0)$ are the saddle points, and $E_{4} (1,1)$ is an unstable point. At this point, (negative guidance, negative response) is the evolutionary stabilization strategy.
2. When ${(}B_{1} - C_{1} - C_{11} + C_{21} {)} - p_{21} {(}B_{1} - C_{1} - C_{11} {)} < 0$,$(B_{3} - C_{21} ) - q_{21} (B_{4} - C_{21} ) > 0$, $E_{2} (0,1)$ is the stable point, $E_{1} (0,0)$ and $E_{4} (1,1)$ are the saddle points, and $E_{3} (1,0)$ is an unstable point. At this time, (negative guidance, positive response) is the evolutionary stabilization strategy.
3. When ${(}B_{1} - C_{1} - C_{11} + C_{21} {)} - p_{21} {(}B_{1} - C_{1} - C_{11} {)} > 0$,$(B_{3} - C_{21} ) - q_{21} (B_{4} - C_{21} ) < 0$, $E_{3} (1,0)$ is the stable point, $E_{1} (0,0)$ and $E_{4} (1,1)$ are the saddle points, and $E_{2} (0,1)$ is an unstable point. At this point, (positive guidance, negative response) is the evolutionary stabilization strategy.
4. When ${(}B_{1} - C_{1} - C_{11} + C_{21} {)} - p_{21} {(}B_{1} - C_{1} - C_{11} {)} > 0$,$(B_{3} - C_{21} ) - q_{21} (B_{4} - C_{21} ) > 0$, $E_{4} (1,1)$ is the stable point, $E_{2} (0,1)$ and $E_{3} (1,0)$ are the saddle points, and $E_{1} (0,0)$ is an unstable point. At this time, (positive guidance, positive response) is the evolutionary stabilization strategy.
5. When $$B_{5} - B_{3} + C_{21} + q_{21} (B_{4} - C_{21} ) - q_{21} B_{6} = 0$$ and $$(1 - p_{21} )(B_{2} + C_{11} - B_{1} ) \ne 0$$, there are four equilibrium points in the evolutionary game model: $$E_{1} (0,0)$$, $$E_{2} (0,1)$$, $$E_{3} (1,0)$$ and $$E_{4} (1,1)$$. The determinant and trace for Jacobian matrices can be discussed in eight cases: (1) $$(B_{3} - C_{21} ) - q_{21} (B_{4} - C_{21} ) < 0$$,$$(B_{2} - C_{1} + C_{21} ) - p_{21} (B_{2} - C_{1} ) < 0$$ and $${(}B_{1} - C_{1} - C_{11} + C_{21} {)} - p_{21} {(}B_{1} - C_{1} - C_{11} {)} < 0$$; (2) $$(B_{2} - C_{1} + C_{21} ) - p_{21} (B_{2} - C_{1} ) < 0$$,$${(}B_{1} - C_{1} - C_{11} + C_{21} {)} - p_{21} {(}B_{1} - C_{1} - C_{11} {)} > 0$$ and $$(B_{3} - C_{21} ) - q_{21} (B_{4} - C_{21} ) < 0$$; (3) $${(}B_{1} - C_{1} - C_{11} + C_{21} {)} - p_{21} {(}B_{1} - C_{1} - C_{11} {)} < 0$$, $$(B_{2} - C_{1} + C_{21} ) - p_{21} (B_{2} - C_{1} ) > 0$$ and $$(B_{3} - C_{21} ) - q_{21} (B_{4} - C_{21} ) < 0$$; (4) $${(}B_{1} - C_{1} - C_{11} + C_{21} {)} - p_{21} {(}B_{1} - C_{1} - C_{11} {)} > 0$$$$(B_{2} - C_{1} + C_{21} ) - p_{21} (B_{2} - C_{1} ) < 0$$, and $$(B_{3} - C_{21} ) - q_{21} (B_{4} - C_{21} ) > 0$$; (5) $${(}B_{1} - C_{1} - C_{11} + C_{21} {)} - p_{21} {(}B_{1} - C_{1} - C_{11} {)} < 0$$, $$(B_{2} - C_{1} + C_{21} ) - p_{21} (B_{2} - C_{1} ) < 0$$ and $$(B_{3} - C_{21} ) - q_{21} (B_{4} - C_{21} ) > 0$$; (6) $${(}B_{1} - C_{1} - C_{11} + C_{21} {)} - p_{21} {(}B_{1} - C_{1} - C_{11} {)} < 0$$$$(B_{2} - C_{1} + C_{21} ) - p_{21} (B_{2} - C_{1} ) > 0$$, and $$(B_{3} - C_{21} ) - q_{21} (B_{4} - C_{21} ) > 0$$; (7) $${(}B_{1} - C_{1} - C_{11} + C_{21} {)} - p_{21} {(}B_{1} - C_{1} - C_{11} {)} > 0$$, $$(B_{2} - C_{1} + C_{21} ) - p_{21} (B_{2} - C_{1} ) > 0$$ and $$(B_{3} - C_{21} ) - q_{21} (B_{4} - C_{21} ) < 0$$; (8) $${(}B_{1} - C_{1} - C_{11} + C_{21} {)} - p_{21} {(}B_{1} - C_{1} - C_{11} {)} > 0$$$$(B_{2} - C_{1} + C_{21} ) - p_{21} (B_{2} - C_{1} ) > 0$$, and $$(B_{3} - C_{21} ) - q_{21} (B_{4} - C_{21} ) > 0$$. The results are shown in Table 3.

**Table 3:** Evolutionary stability analysis of the model ($B_{5} - B_{3} + C_{21} + q_{21} (B_{4} - C_{21} ) - q_{21} B_{6} = 0$, $(1 - p_{21} )(B_{2} + C_{11} - B_{1} ) \ne 0$).

Case	Basis of Judgment	(0,0)	(0,1)	(1,0)	(1,1)
Case 1	$\det J$	+	−	−	+
	$trJ$	−	Uncertainty	Uncertainty	+
	Stability	ESS	Saddle point	Saddle point	Unstable point
Case 2	$\det J$	+	+	−	−
	$trJ$	−	+	Uncertainty	Uncertainty
	Stability	ESS	Unstable point	Saddle point	Saddle point
Case 3	$\det J$	−	−	+	+
	$trJ$	Uncertainty	Uncertainty	−	+
	Stability	Saddle point	Saddle point	ESS	Unstable point
Case 4	$\det J$	−	−	+	+
	$trJ$	Uncertainty	Uncertainty	+	−
	Stability	Saddle point	Saddle point	Unstable point	ESS
Case 5	$\det J$	−	+	+	−
	$trJ$	Uncertainty	−	+	Uncertainty
	Stability	Saddle point	ESS	Unstable point	Saddle point
Case 6	$\det J$	+	+	−	−
	$trJ$	+	−	Uncertainty	Uncertainty
	Stability	Unstable point	ESS	Saddle point	Saddle point
Case 7	$\det J$	−	+	+	−
	$trJ$	Uncertainty	+	−	Uncertainty
	Stability	Saddle point	Unstable point	ESS	Saddle point
Case 8	$\det J$	+	−	−	+
	$trJ$	+	Uncertainty	Uncertainty	−
	Stability	Unstable point	Saddle point	Saddle point	ESS

The other two cases are shown in the Appendix A.

(III) See Appendix A.6.1.

(IV) See Appendix A.6.2.

### Stability analysis of the dynamic delayed SEIR evolutionary game model

It should be noted that the recovered equations are independent in Model (15) and have no influence on the stability analysis, so Model (15) can be decoupled to obtain the following delayed model:

19 $$\left\{ \begin{gathered} \frac{dS}{{dt}} = a - \frac{\beta SI(t - \tau )}{{1 + \alpha I(t - \tau )}} - mx_{1} S \hfill \\ \frac{dE}{{dt}} = \frac{\beta SI(t - \tau )}{{1 + \alpha I(t - \tau )}} - mx_{2} E - y_{1} E + y_{2} I \hfill \\ \frac{dI}{{dt}} = mx_{2} E - \gamma I - y_{2} I \hfill \\ \end{gathered} \right.$$

Regarding Model (19), if $R < 1$, infection-free equilibrium $E^{0} = (\frac{a}{{mx_{1} }}0,0)$ always exists. If $R > 1$, this model has only positive equilibrium, namely, $E^{e} = (S^{ * } ,E^{ * } ,I^{ * } )$.

Here, $$S^{ * } = \frac{{1 + \alpha I^{ * } }}{\beta }(\frac{\beta a}{{Rmx_{1} }} - y_{2} )$$, $$E^{ * } = \frac{{(\gamma + y_{2} )}}{{mx_{2} }}I^{ * }$$ and $$I^{ * } = \frac{{1 - (\frac{1}{R} - \frac{{mx_{1} y_{2} }}{\beta a})}}{{(\frac{\beta }{{Rmx_{1} }} - \frac{{y_{2} }}{a})(1 + \frac{{\alpha mx_{1} }}{\beta })}}$$.

The basic reproduction number can be computed as follows ^41^:

$$R = \frac{{\beta ax_{2} }}{{x_{1} (mx_{2} + y_{1} )(\gamma + y_{2} )}}$$

#### Stability analysis of no infection equilibrium

Theorem 3.1. The infection-free equilibrium $E^{0}$ is locally asymptotically stable if $R < 1$.

##### ***Proof***

The corresponding characteristic equation of Model (19) at $E^{0}$ is.

20 $$(\lambda + mx_{1} )\{ [\lambda + (mx_{2} + y_{1} )][\lambda + (\gamma + y_{2} )] - y_{2} \beta S^{0} mx_{2} e^{ - \lambda \tau } \} = 0$$

According to (20), we obtain the eigenvalue $\lambda = - mx_{1} < 0$.The other eigenvalues of (21) can be rewritten as

21 $$[\lambda + (mx_{2} + y_{1} )][\lambda + (\gamma + y_{2} )] - y_{2} \beta S^{0} mx_{2} e^{ - \lambda \tau } = 0$$

There are two situations for discussion:

(i) If $\tau = 0$, Eq. (21) can be written in the following form:
  22 $$[\lambda + (mx_{2} + y_{1} )][\lambda + (\gamma + y_{2} )] - y_{2} \beta S^{0} mx_{2} = 0$$

Equation (22) can be written as the following equation

23 $$\lambda^{2} + A\lambda + B{ = }0$$

where $$A = mx_{2} + y_{1} + \gamma + y_{2}$$, $$B = (mx_{2} + y_{1} )(\gamma + y_{2} ) - \frac{{y_{2} \beta ax_{2} }}{{x_{1} }}$$。

If $R < 1$, so $A > 0$, $B > 0$. All eigenvalues of the Eq. (23) have negative roots. Therefore, the infection-free equilibrium, $E^{0}$, is locally asymptotically stable.

(ii) Let $\tau = i\omega (\omega > 0)$, substituting for in the Eq. (21), separating real and imaginary parts, we can obtain
  24 $$\left\{ {\begin{array}{*{20}l} {\frac{{y_{2} \beta ax_{2} }}{{x_{1} }}\cos (\omega \tau ) = mx_{2} + y_{1} + \gamma + y_{2} } \hfill \\ { - \frac{{y_{2} \beta ax_{2} }}{{x_{1} }} \cdot {\text{i}}\sin (\omega \tau ) = {\text{i}}\omega } \hfill \\ \end{array} } \right.$$

We square and add the two equations of (24), yielding

25 $$\omega^{2} = (\frac{{y_{2} \beta ax_{2} }}{{x_{1} }})^{2} - (mx_{2} + y_{1} + \gamma + y_{2} )^{2}$$

If $R < 1$, we get

26 $$(\frac{{y_{2} \beta ax_{2} }}{{x_{1} }})^{2} < (mx_{2} + y_{1} + \gamma + y_{2} )^{2}$$

Thus, $\omega^{2} < 0$ which is a contradiction, so the roots have negative real parts.

In summary, if $R < 1$, the infection-free equilibrium $E^{0}$ is locally asymptotically stable for all $\tau \ge 0$.

Theorem 3.2. The infection-free equilibrium $E^{0}$ is globally asymptotically stable if $R < 1$ and $\gamma > \frac{\beta a}{{mx_{1} }}$.

##### ***Proof***

We choose the Lyapunov function.

27 $$L_{1} { = }S - S^{0} - S\ln \frac{S}{{S^{0} }} + E + I + \int_{t - \tau }^{t} {\frac{{\beta S^{0} I(s)}}{1 + \alpha I(s)}ds}$$

Then,

28 $$\begin{gathered} \frac{{dL_{1} }}{dt}{ = }\frac{{S - S^{0} }}{{S^{0} }}(a - \frac{\beta SI(t - \tau )}{{1 + \alpha I(t - \tau )}} - mx_{1} S) + \frac{\beta SI(t - \tau )}{{1 + \alpha I(t - \tau )}} - mx_{2} E - y_{1} E + y_{2} I \hfill \\ {\kern 1pt} {\kern 1pt} {\kern 1pt} {\kern 1pt} {\kern 1pt} {\kern 1pt} {\kern 1pt} {\kern 1pt} {\kern 1pt} {\kern 1pt} {\kern 1pt} {\kern 1pt} {\kern 1pt} {\kern 1pt} {\kern 1pt} {\kern 1pt} {\kern 1pt} {\kern 1pt} {\kern 1pt} {\kern 1pt} {\kern 1pt} {\kern 1pt} {\kern 1pt} + mx_{2} E - \gamma I - y_{2} I + \frac{{\beta S^{0} I}}{1 + \alpha I} - \frac{{\beta S^{0} I(t - \tau )}}{1 + \alpha I(t - \tau )} \hfill \\ {\kern 1pt} {\kern 1pt} {\kern 1pt} {\kern 1pt} {\kern 1pt} {\kern 1pt} {\kern 1pt} {\kern 1pt} {\kern 1pt} {\kern 1pt} {\kern 1pt} {\kern 1pt} {\kern 1pt} {\kern 1pt} {\kern 1pt} = - \frac{{mx_{1} }}{{S^{0} }}(S - S^{0} )^{2} - y_{1} E - \gamma I + \frac{{\beta S^{0} I}}{1 + \alpha I} \hfill \\ {\kern 1pt} {\kern 1pt} {\kern 1pt} {\kern 1pt} {\kern 1pt} {\kern 1pt} {\kern 1pt} {\kern 1pt} {\kern 1pt} {\kern 1pt} {\kern 1pt} {\kern 1pt} {\kern 1pt} {\kern 1pt} {\kern 1pt} = - \frac{{mx_{1} }}{{S^{0} }}(S - S^{0} )^{2} - y_{1} E - I(\gamma - \frac{\beta a}{{mx_{1} }}) < 0 \hfill \\ \end{gathered}$$

Since $\{ (S,E,I) \in R_{ + }^{3} :\frac{{dL_{1} }}{dt} = 0\} = \{ (S,E,I) \in R_{ + }^{3} :S = S^{0} = \frac{a}{{mx_{1} }},E = E^{0} = 0,I = I^{0} = 0\}$. Combined with the LaSalle invariance principle ^42^, the infection-free equilibrium $E^{0}$ is globally asymptotically stable.

#### Stability analysis of positive equilibrium

Theorem 3.3. The positive equilibrium $E^{e}$ is locally asymptotically stable if $R > 1$.

##### ***Proof***

The corresponding characteristic equation of Model (19) at $E^{e}$ is.

29 $$\left| {\begin{array}{*{20}c} {\lambda + \frac{{\beta I^{ * } }}{{1 + \alpha I^{ * } }} + mx_{1} } & 0 & {\frac{{\beta S^{ * } }}{{(1 + \alpha I^{ * } )^{2} }}e^{ - \lambda \tau } } \\ { - \frac{{\beta I^{ * } }}{{1 + \alpha I^{ * } }}} & {\lambda + (mx_{2} + y_{1} )} & { - y_{2} - \frac{{\beta S^{ * } }}{{(1 + \alpha I^{ * } )^{2} }}e^{ - \lambda \tau } } \\ 0 & { - mx_{2} } & {\lambda + (\gamma + y_{2} )} \\ \end{array} } \right| = 0$$

That is

30 $$\begin{gathered} {\kern 1pt} {\kern 1pt} {\kern 1pt} {\kern 1pt} {\kern 1pt} {\kern 1pt} {\kern 1pt} {\kern 1pt} {\kern 1pt} \lambda^{3} + (b + c + a + mx_{1} )\lambda^{2} + [bc - mx_{2} + (a + mx_{1} )(b + c)]\lambda \hfill \\ + (a + mx_{1} )(bc - mx_{2} y_{2} ) + [admx_{2} - dmx_{2} \lambda - (a + mx_{1} )dmx_{2} ]e^{ - \lambda \tau } = 0 \hfill \\ \end{gathered}$$

where $$a = \frac{{\beta I^{ * } }}{{1 + \alpha I^{ * } }}$$, $$b = mx_{2} + y_{1}$$, $$c = \gamma + y_{2}$$, $$d{ = }\frac{{\beta S^{ * } }}{{(1 + \alpha I^{ * } )^{2} }}$$.

Two situations were discussed:

(i) Equation (30) with $\tau = 0$ is written in the following form:
  31 $$a_{0} \lambda^{3} + a_{1} \lambda^{2} + a_{2} \lambda + a_{3} = 0$$

where

$$a_{0} = 1$$, $$a_{1} = b + c + a + mx_{1} > 0$$, $$a_{2} = bc - mx_{2} y_{2} + (a + mx_{1} )(b + c) - mx_{2} d$$,

$$a_{3} = (a + mx_{1} )(bc - mx_{2} y_{2} ) + admx_{2} - (a + mx_{1} )dmx_{2}$$。

Then,

32 $$\begin{aligned} a_{2} = & bc - mx_{2} y_{2} + (a + mx_{1} )(b + c) - mx_{2} d \\ {\kern 1pt} & {\kern 1pt} = (mx_{2} + y_{1} )(\gamma + y_{2} )(1 - \frac{1}{{1 + \alpha I^{ * } }}) + \frac{{mx_{2} y_{2} }}{{1 + \alpha I^{ * } }} + \frac{{\beta I^{ * } }}{{1 + \alpha I^{ * } }}(mx_{2} + 1 + \gamma ) \\ & {\kern 1pt} + mx_{1} (mx_{2} + y_{1} + \gamma ) + m(x_{1} - x_{2} )y_{2} > 0 \\ \end{aligned}$$

33 $$\begin{gathered} a_{3} = (a + mx_{1} )(bc - mx_{2} y_{2} ) + admx_{2} - (a + mx_{1} )dmx_{2} \hfill \\ {\kern 1pt} {\kern 1pt} {\kern 1pt} {\kern 1pt} {\kern 1pt} {\kern 1pt} {\kern 1pt} {\kern 1pt} {\kern 1pt} {\kern 1pt} {\kern 1pt} = (a + mx_{1} )\{ (mx_{2} \gamma + y_{1} (\gamma + y_{2} )](1 - \frac{1}{{1 + \alpha I^{ * } }})\} + admx_{2} > 0 \hfill \\ \end{gathered}$$

It follows from the Routh-Hurwitz criteria ^43^ that all roots of the characteristic Eq. (31) have negative real parts.

(ii) (*ii*) $\tau = i\omega (\omega > 0)$ is assumed to be a root of Eq. (30). Then, $\omega$ should satisfy the following equation:
  34 $$\begin{gathered} {\kern 1pt} {\kern 1pt} {\kern 1pt} {\kern 1pt} {\kern 1pt} {\kern 1pt} {\kern 1pt} {\kern 1pt} (i\omega )^{3} + (b + c + a + mx_{1} ){\kern 1pt} {\kern 1pt} (i\omega )^{2} + [bc - mx_{2} + (a + mx_{1} )(b + c)]{\kern 1pt} {\kern 1pt} (i\omega ) \hfill \\ + (a + mx_{1} )(bc - mx_{2} y_{2} ) + [admx_{2} - dmx_{2} {\kern 1pt} (i\omega ) - (a + mx_{1} )dmx_{2} ]e^{{ - {\kern 1pt} {\kern 1pt} (i\omega )\tau }} = 0 \hfill \\ \end{gathered}$$

Separating real and imaginary parts implies that

35 $$\left\{ {\begin{array}{*{20}l} \begin{aligned} - \omega^{2} (b + c + a + mx_{1} ) + (a + mx_{1} )(bc - mx_{2} y_{2} ) = & dmx_{2} \omega \sin \omega \tau \\ & {\kern 1pt} - [admx_{2} - (a + mx_{1} )dmx_{2} ]{\kern 1pt} {\kern 1pt} \cos \omega \tau \\ \end{aligned} \hfill \\ \begin{aligned} - \omega^{3} + [bc - mx_{2} + (a + mx_{1} )(b + c)]{\kern 1pt} {\kern 1pt} \omega = {\kern 1pt} & {\kern 1pt} {\kern 1pt} dmx_{2} \omega \cos \omega \tau \\ {\kern 1pt} & {\kern 1pt} {\kern 1pt} + [admx_{2} - (a + mx_{1} )dmx_{2} ]{\kern 1pt} {\kern 1pt} \sin \omega \tau \\ \end{aligned} \hfill \\ \end{array} } \right.$$

Taking square on both sides of the equations of (35) and summing them up, we obtain

36 $$Z^{3} + b_{1} Z^{3} + b_{2} Z^{3} + b_{3} { = }0$$

where

$$Z = \omega^{2}$$,

$$\begin{gathered} b_{1} { = }(b + c + a + mx_{1} )^{2} - 2[bc - mx_{2} + (a + mx_{1} )(b + c)] \hfill \\ {\kern 1pt} {\kern 1pt} {\kern 1pt} {\kern 1pt} {\kern 1pt} {\kern 1pt} {\kern 1pt} {\kern 1pt} = (a + mx_{1} )^{2} + 2mx_{2} + b^{2} + c^{2} > 0 \hfill \\ \end{gathered}$$,

$$\begin{gathered} b_{2} = [bc - mx_{2} + (a + mx_{1} )(b + c)]{\kern 1pt} {\kern 1pt}^{2} - 2(b + c + a + mx_{1} )(a + mx_{1} )(bc - mx_{2} y_{2} ) - (dmx_{2} )^{2} \hfill \\ {\kern 1pt} {\kern 1pt} {\kern 1pt} {\kern 1pt} {\kern 1pt} {\kern 1pt} {\kern 1pt} {\kern 1pt} {\kern 1pt} {\kern 1pt} {\kern 1pt} {\kern 1pt} \ge {\kern 1pt} {\kern 1pt} {\kern 1pt} b^{2} c^{2} (1 - \frac{1}{{1 + \alpha I^{ * } }}) + m^{2} x_{2}^{2} (1 - \frac{1}{{1 + \alpha I^{ * } }}) + 2bcm\frac{1}{{1 + \alpha I^{ * } }} + 2mx_{2} (a + mx_{1} )^{2} + 2abc(a + mx_{1} + m) \hfill \\ \end{gathered}$$

$$\begin{gathered} b_{3} { = }(a + mx_{1} )^{2} (bc - mx_{2} y_{2} )^{2} - [admx_{2} - (a + mx_{1} )dmx_{2} ]{\kern 1pt} {\kern 1pt}^{2} \hfill \\ {\kern 1pt} {\kern 1pt} {\kern 1pt} {\kern 1pt} {\kern 1pt} {\kern 1pt} {\kern 1pt} {\kern 1pt} {\kern 1pt} = \{ (a + mx_{1} )^{2} [mx_{2} \gamma + y(\gamma + y_{2} )]^{2} - d^{2} m^{2} x_{1} x_{2} \hfill \\ \end{gathered}$$

Because the equilibrium point of the evolutionary game needs to satisfy the Nash equilibrium, the value of $x_{1} ,x_{2} ,y_{1} ,y_{2}$ is 1, 0. Moreover, based on assumption 4, we know $x_{1} x_{2} = 0$ and then, we obtain $b_{1} > 0,b_{2} > 0,b_{3} > 0$. Thus, all roots of Eq. (34) have no positive real roots and $\omega^{2}$ is meaningless. The characteristic equation has no pure imaginary roots, and all roots of the characteristic equation have negative real parts at $\tau > 0$.

Theorem 3.4. The positive equilibrium $E^{e}$ is globally asymptotically stable.

##### ***Proof***

In accordance with the literature ^14^, the Lyapunov function is constructed:

37 $$L_{2} = [(S - S^{ * } ) + (E - E^{ * } ) + (I - I^{ * } )]^{2}$$

Then,

38 $$\begin{gathered} \frac{{dL_{2} }}{dt} = 2[(S - S^{ * } ) + (E - E^{ * } ) + (I - I^{ * } )](S^{\prime} + E^{\prime} + I^{\prime}) \hfill \\ {\kern 1pt} {\kern 1pt} {\kern 1pt} {\kern 1pt} {\kern 1pt} {\kern 1pt} {\kern 1pt} {\kern 1pt} {\kern 1pt} {\kern 1pt} {\kern 1pt} {\kern 1pt} {\kern 1pt} {\kern 1pt} {\kern 1pt} {\kern 1pt} {\kern 1pt} {\kern 1pt} {\kern 1pt} {\kern 1pt} {\kern 1pt} = 2[(S - S^{ * } ) + (E - E^{ * } ) + (I - I^{ * } )](a - mx_{1} S - y_{1} E - \gamma I) \hfill \\ {\kern 1pt} {\kern 1pt} {\kern 1pt} {\kern 1pt} {\kern 1pt} {\kern 1pt} {\kern 1pt} {\kern 1pt} {\kern 1pt} {\kern 1pt} {\kern 1pt} {\kern 1pt} {\kern 1pt} {\kern 1pt} {\kern 1pt} {\kern 1pt} {\kern 1pt} {\kern 1pt} {\kern 1pt} {\kern 1pt} = 2[(S - S^{ * } ) + (E - E^{ * } ) + (I - I^{ * } )][mx_{1} (S^{ * } - S) + y_{1} (E^{ * } - E) + \gamma (I^{ * } - I)] \hfill \\ {\kern 1pt} {\kern 1pt} {\kern 1pt} {\kern 1pt} {\kern 1pt} {\kern 1pt} {\kern 1pt} {\kern 1pt} {\kern 1pt} {\kern 1pt} {\kern 1pt} {\kern 1pt} {\kern 1pt} {\kern 1pt} {\kern 1pt} {\kern 1pt} {\kern 1pt} {\kern 1pt} {\kern 1pt} \le - 2[mx_{1} (S - S^{ * } )^{2} + y_{1} (E - E^{ * } )^{2} + \gamma (I - I^{ * } )^{2} ] \le 0 \hfill \\ \end{gathered}$$

Therefore, based on LaSalle invariance principle ^42^, we obtain that the positive equilibrium $E^{e}$ is globally asymptotically stable.

## Numerical simulation

Let $B_{1} = 15$, $B_{2} = 10$, $C_{1} = 5$, $C_{21} = 4$, $C_{11} = 5$, $B_{3} = 7$, $B_{4} = 5$, $B_{5} = 3$, $B_{6} = 1$, $\lambda_{1} = 1$, $\rho_{1} = 1$, $a = 1$, $\beta = 0.2$, $\gamma = 0.5$, $\alpha = 1$, $m = 0.5$. The initial value of the improving evolutionary game model is $({0}{\text{.6,0}}{.5})$. The initial value of the dynamic delayed SEIR evolutionary game model is $({2,8,10})$.

### Numerical simulation of the improved evolutionary game model

Various dependency coefficients are selected to simulate the evolutionary process of the strategies of the evolutionary game model. In real life, it is necessary to avoid the negative impact of emergencies and to maintain social stability. The official opinion field actively guides, while the opinion field adopts a positive response. Therefore, only the case where stable equilibrium strategies are (1,1) is modelled, i.e., "positive guidance, positive response".

**Figure 1:**
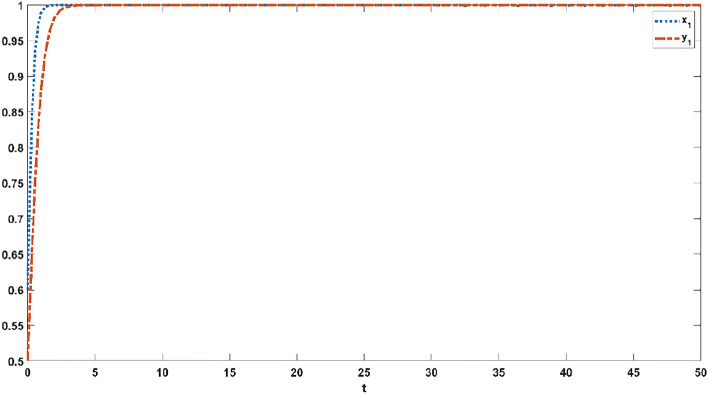
If $p21 = 1,q21 = 1$, the evolution process of strategies in the official opinion field and the public opinion field.

**Figure 2:**
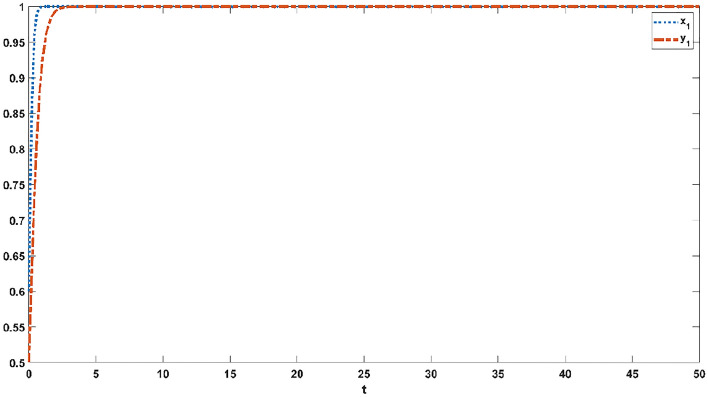
If $p21 = 0.5,q21 = 0.5$, the evolution process of strategies in the official opinion field and the public opinion field.

**Figure 3:**
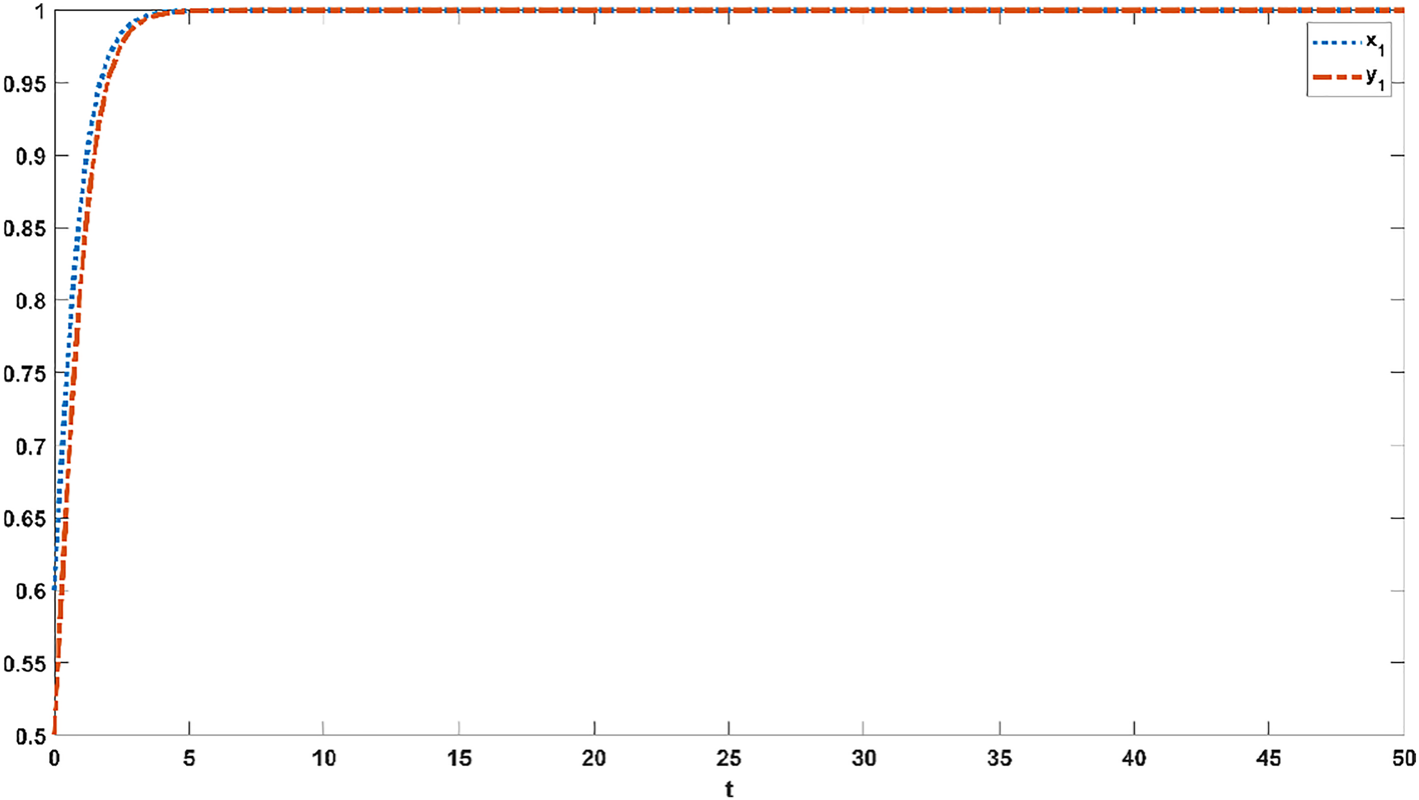
If $p21 = 1.5,q21 = 1.5$, the evolution process of strategies in the official opinion field and the public opinion field.

**Figure 4:**
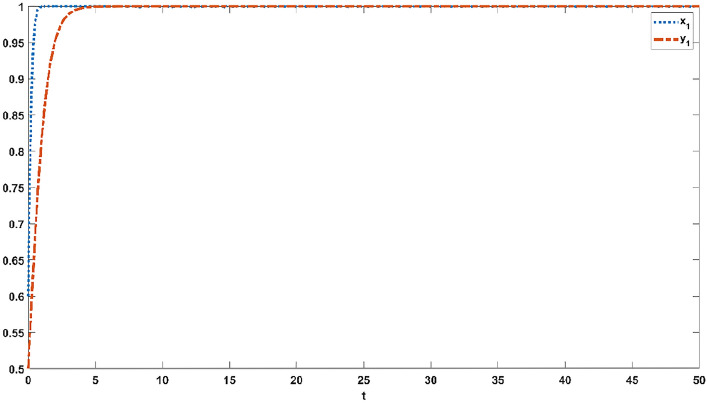
If $p21 = 0.5,q21 = 1.5$, the evolution process of strategies in the official opinion field and the public opinion field.

**Figure 5:**
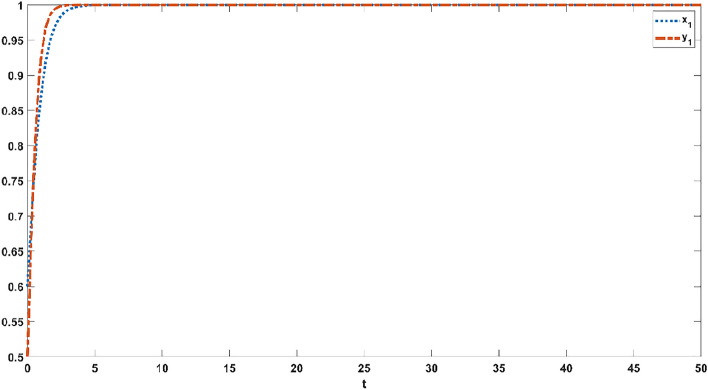
If $p21 = 1.5,q21 = 0.5$, the evolution process of strategies in the official opinion field and the public opinion field.

Figures 1, 2, 3, 4 and 5 simulate the evolutionary process of strategies of the improving evolutionary game model under the various dependency coefficients. The blue curve represents the evolution process of strategies in the official opinion field, while the orange curve represents the evolution process of strategies in the public opinion field.

The dependence coefficients are $p21 = 1,q21 = 1$ in Fig. 1. When the dependence coefficient is 1, the model uses the traditional replicator dynamic. The official opinion field reaches a steady state at 15 s, while the public opinion field reaches a steady state at 23 s. Then, the model reaches a steady state at 23 s.

Figures 2 and 3 simulate the evolutionary process of strategies in the official opinion field and the public opinion field when the coefficient of dependence of the two game groups is less than 1 and greater than 1, respectively. As shown in Fig. 2, the official opinion field reaches a steady state at 14 s, and the public opinion field reaches a steady state at 22 s. Moreover, the two opinion fields reached a steady state at 26 s in Fig. 3. Comparing Fig. 2 with Figs. 1 and 3, the time needed to reach a steady state was reduced. This suggests that there is a positive effect on two strategies in the same game group (i.e., the official opinion field and the public opinion field), accelerating the speed of game convergence.

When $p21 = 0.5,q21 = 1.5$, the official opinion field reaches a steady state at 19 s, and the public opinion field reaches a steady state at 29 s, as shown in Fig. 4. When $p21 = 1.5,q21 = 0.5$, Fig. 5 indicates that the official opinion field reaches a steady state at 26 s and that the public opinion field reaches a steady state at 21 s. Different values of the dependence coefficient have different effects on the speed of the game convergence, as shown in Figs. 2, 3, 4 and 5. When the dependence coefficient is less than 1, the different strategies within the game group have a positive effect and accelerate the speed of the game convergence, while when the dependence coefficient is greater than 1, the different strategies within the game group have a negative effect and slow down the speed of the game convergence. Therefore, it is concluded that the strategy dependency among the same game group has positive and negative effects on the evolution process.

### Numerical simulation of the delayed panic SEIR spread model

The evolution process of the dynamic delayed panic SEIR evolutionary game spread model under the effect of a positive effect is simulated in this section.

**Figure 6:**
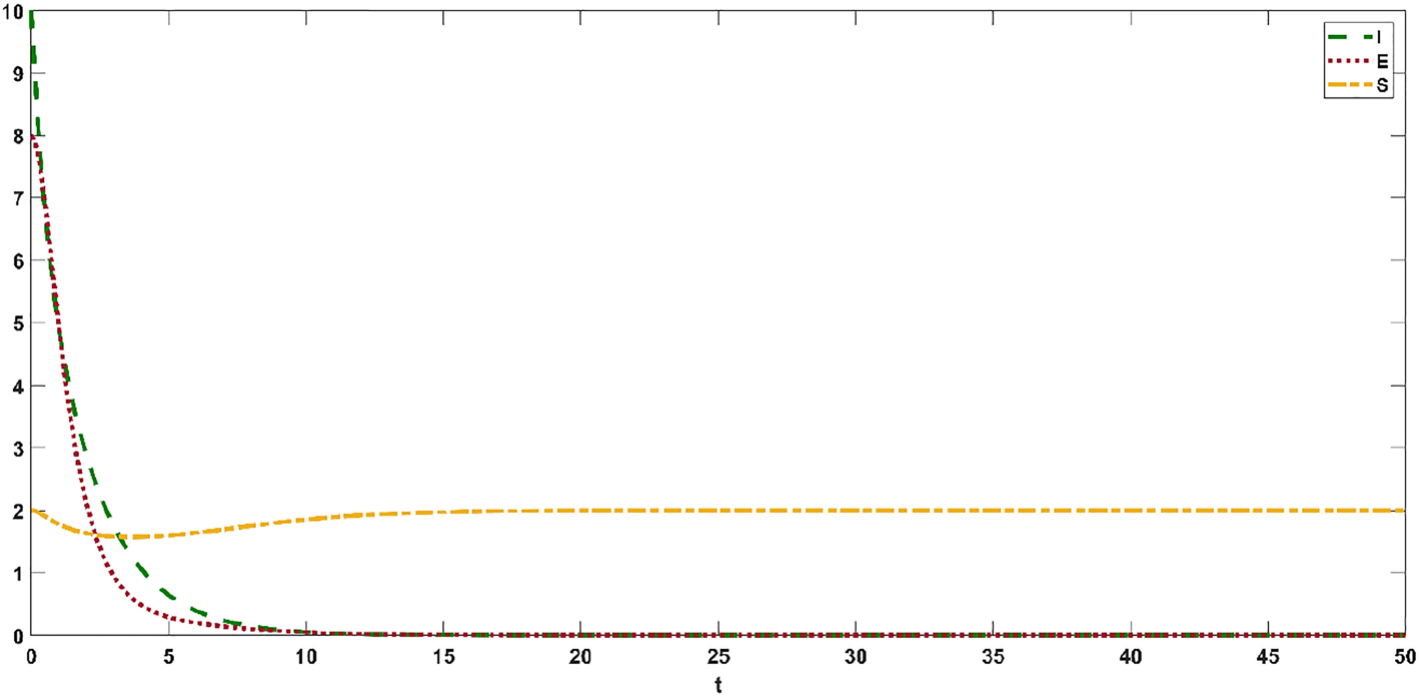
Evolution trend of the dynamic delayed panic SEIR evolutionary game model.

The cases where $R < 1$ and dependency coefficient $p21 = 0.5,q21 = 0.5$ are shown in Fig. 6, which shows the evolution trend of infected individuals, exposed individuals and susceptible individuals. As shown in Fig. 6, in the initial stage of panic contagion, the infected individuals and exposed individuals decrease sharply in a short time, and the susceptible individuals first decrease and then increase. As time varied, the delayed model reached a stable state. This shows that when an emergency occurs, the official public opinion field and the public opinion field adopt the strategy of "positive guidance and positive response" to make the infected individuals and exposed individuals in the group disappear, which can control the spread scale of infected individuals and exposed individuals and can reduce the negative consequences of the emergency.

**Figure 7:**
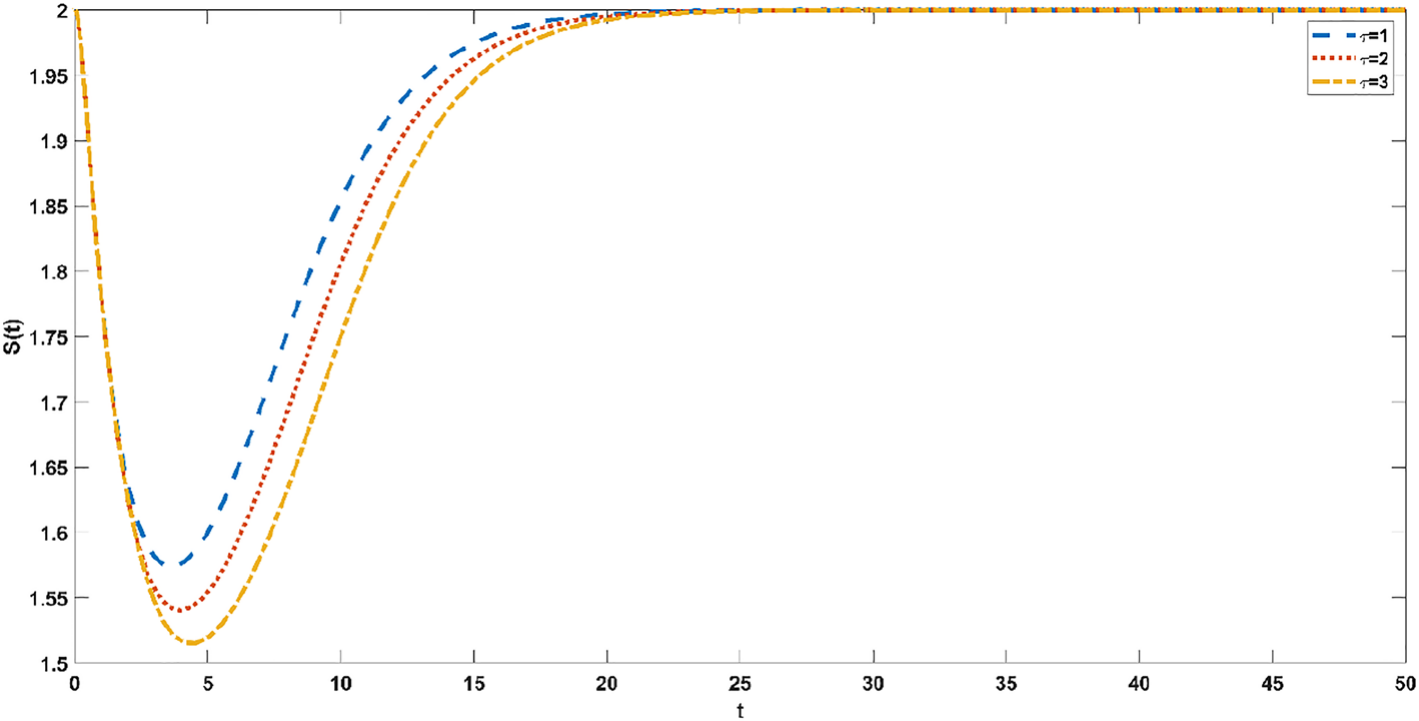
Evolution trend of susceptible individuals with different time delays $\tau = 1,2,3$.

**Figure 8:**
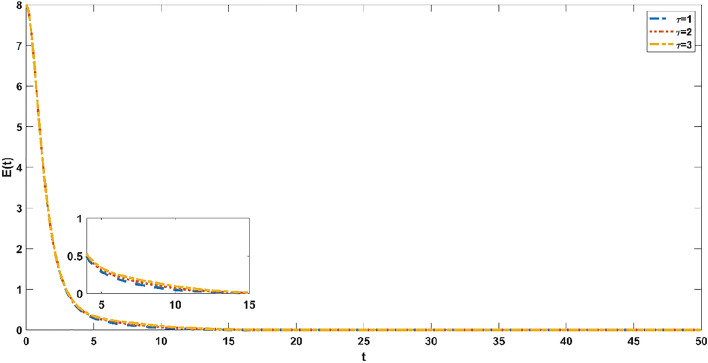
Evolution trend of exposed individuals with different time delays $\tau = 1,2,3$.

Since it takes a period for susceptible individuals to acquire infectious ability after transforming into exposed individuals, it is necessary to investigate the evolution of susceptible persons and incubators at different time delays $\tau = 1,2,3$. Figure 7 simulates the evolution process of the susceptible individuals at different delays. The trend of the susceptible individuals first decreases and then increases, and finally reaches a steady state. As the delay increases, the peak decreases. The trend of exposed individuals decreases dramatically over a short time and eventually reaches a steady state. As the delay increases, so does the time required to reach a steady state, as shown in Fig. 8. As obtained from Figs. 7 and 8, the reduction in delay can accelerate the time for the model to reach a stable state. Therefore, when an emergency occurs, the official opinion field and the public opinion field should respond for the first time to shorten the delay, so that people can quickly understand the truth and progress of the emergency.

### Sensitivity analysis

In this section, sensitivity analysis is performed on the basic regeneration number *R* to explore the effect of the parameters on the model, because the basic regeneration number is an important standard to measure whether panic exists within the group. When the basic regeneration number is less than 1, panic disappears; when the basic regeneration number is greater than 1, panic always exists within the group. To explore the response strategies to panic within a group, relationship between $R$ and parameters *m* and $\gamma$, relationship between $R$ and parameters $\beta$ and *m*, and relationship between $\beta$ and parameters $\gamma$ were simulated, respectively.

**Figure 9:**
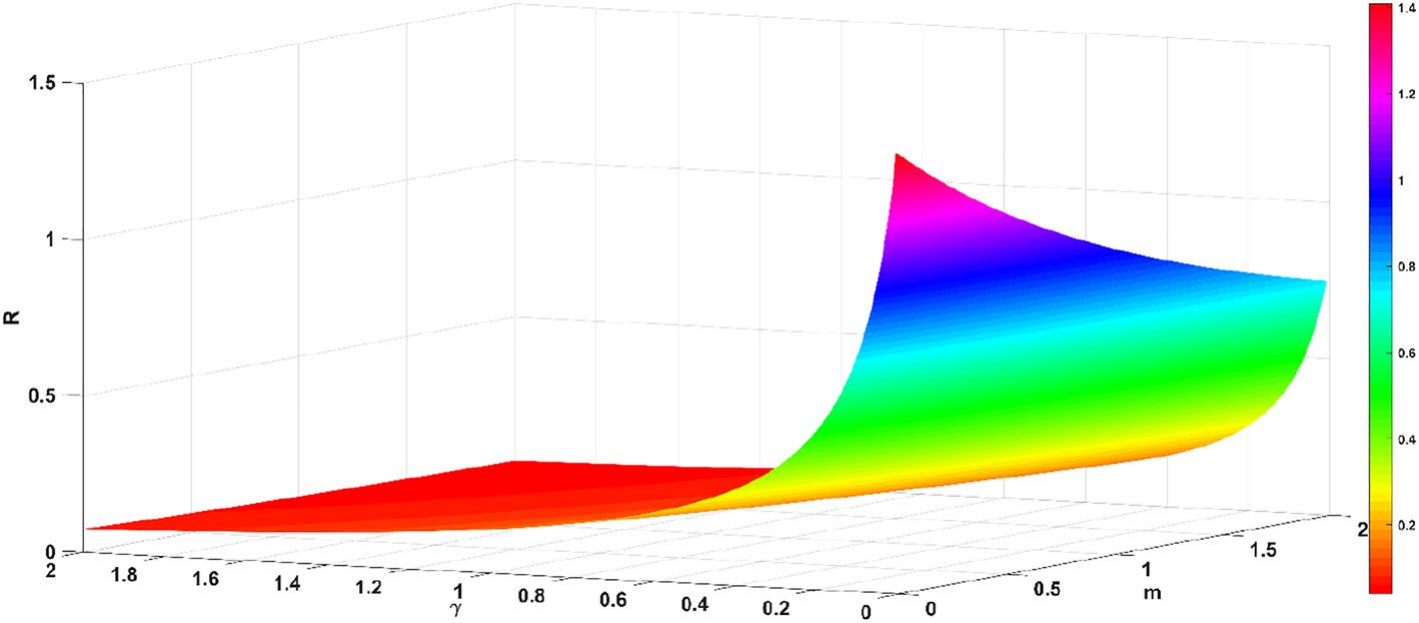
Relationship between $R$ and parameters *m* and $\gamma$.

**Figure 10:**
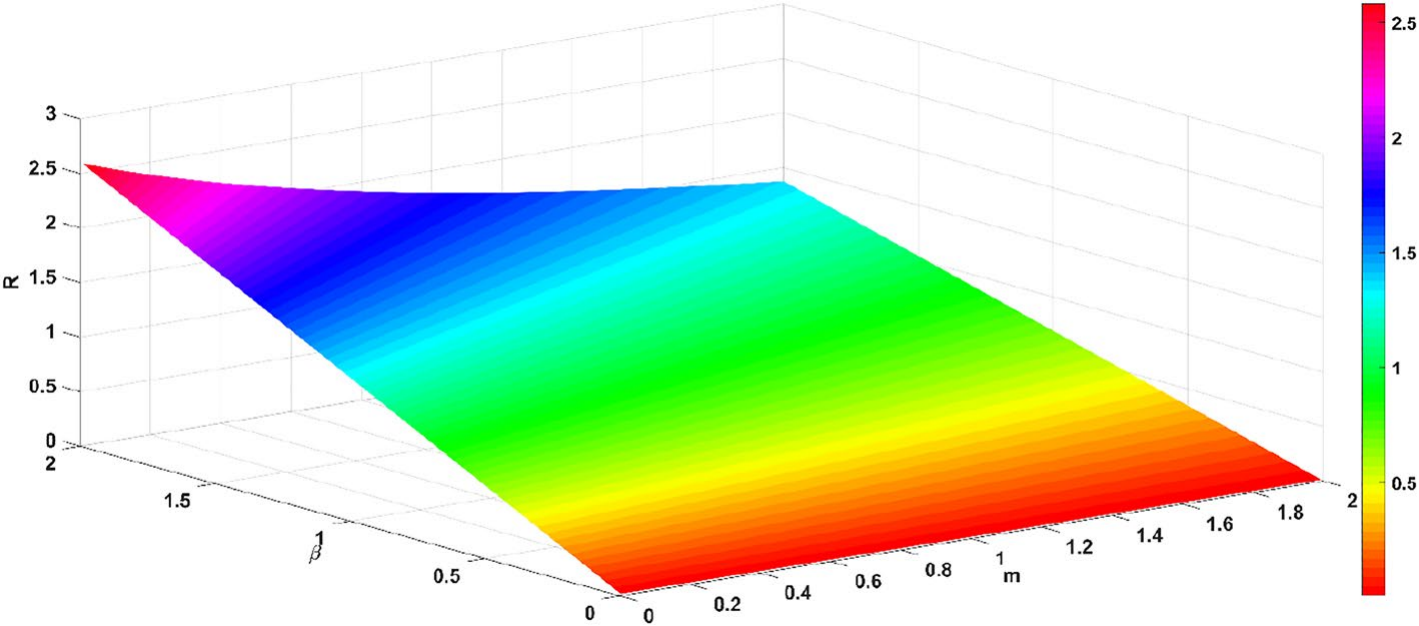
Relationship between $R$ and parameters $\beta$ and *m*.

**Figure 11:**
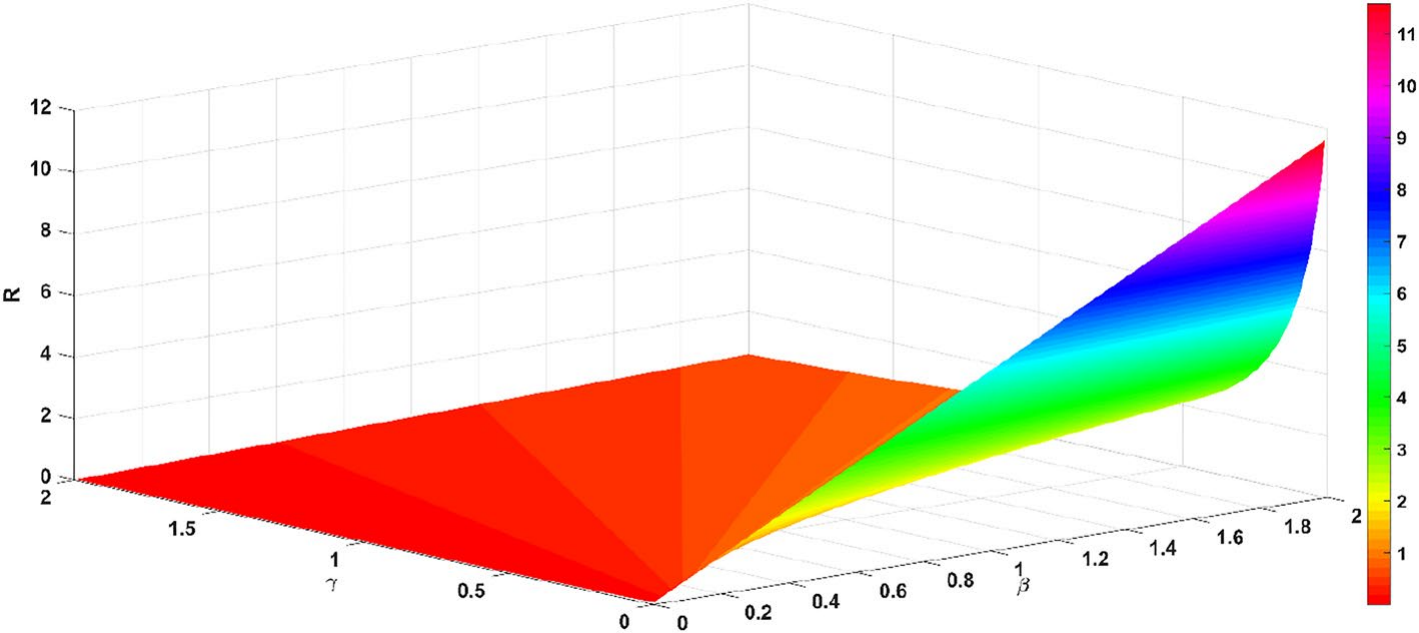
Relationship between $R$ and parameters $\beta$ and $\gamma$.

The basic regeneration number *R* decreases with the increase in the official opinion field authority *m* and the recovery rate $\gamma$, as shown in Fig. 9. The basic regeneration number *R* decreases with the increase in the official opinion field authority *m* and the recovery rate $\gamma$, as shown in Fig. 9. The basic regeneration number *R* increases with the infection rate $\beta$, and decreases with the official opinion field authority *m*, as shown in Fig. 10. The basic regeneration number *R* increases with the infection rate $\beta$, and decreases with the recovery rate $\gamma$, as shown in Fig. 11.

From Figs. 9, 10 and 11, the official opinion field authority *m* and the recovery rate $\gamma$ are able to eliminate the group panic; while the infection rate $\beta$ accelerates the spread of panic within the group. When an emergency occurs, the official opinion field and the public opinion field take measures (timely releasing information and communication and collaboration). As the public recognizes the authority of the official opinion field, while the public opinion field actively cooperates, there is a high probability that individuals in the group will be transformed into recovered individuals. The official opinion field must actively guide the incident at the first time of the emergency, publish the truth and progress of the emergency, and eliminate the negative impact of the emergency, which precisely shows that the measures taken by the official court of public opinion and the private court of public opinion are effective.

## Conclusion

The occurrence of emergencies usually leads to the spread of negative emotions within groups, so it is necessary to analyse the transmission mechanism of panic as well as the decision behaviour of the opinion field. The evolutionary game model of the official opinion field and the public opinion field is established in this paper, while the dependence coefficient is introduced to describe the dependence relationship between the strategies of the same game to improve the traditional replicated dynamic equations. Meanwhile, because the influence of the official opinion field and the public opinion field on panic spread under emergencies is different, that influence is introduced as a time-varying variable into the epidemic model, and the dynamic delayed SEIR evolutionary game model is established, which considers the delayed effects of susceptible and exposed individuals. The equilibrium of the model is analysed for stability. The numerical results show that when the dependence coefficient is less than 1, the different strategies within the same game group accelerate the speed of game convergence due to the positive effect; in contrast, such differences reduce the speed of game convergence.

The aim of this paper is to develop a model for simulating the spread process of panic and the decision behaviour of the opinion field. The results of this research are in line with the changing trends of real situations. In the future, we will compare the results with literature in the field. When an emergency occurs, the official opinion field and the public opinion field must respond to the emergency for the first time, reduce the negative impact, make the panic disappear from the group, and provide the theoretical basis for the relevant the official opinion field emergency departments to make timely and effective decisions and measures. Therefore, it is worth exploring the transmission mechanism for panic spread and the decision behaviour of the opinion field in groups.

## Data Availability

All data analysed during this study are included in this published article.

## Appendix A

In the appendix, we give the analysis of case (III) and case (IV).

## Appendix A 0.6.1

(III) When $B_{5} - B_{3} + C_{21} + q_{21} (B_{4} - C_{21} ) - q_{21} B_{6} \ne 0$ and $(1 - p_{21} )(B_{2} + C_{11} - B_{1} ) = 0$, The four equilibrium points of the evolutionary game model are: $E_{1} (0,0)$, $E_{2} (0,1)$, $E_{3} (1,0)$ and $E_{4} (1,1)$. The determinant and trace for Jacobian matrices should be discussed in eight cases: (1) ${(}B_{1} - C_{1} - C_{11} + C_{21} {)} - p_{21} {(}B_{1} - C_{1} - C_{11} {) < 0}$, $(B_{3} - C_{21} ) - q_{21} (B_{4} - C_{21} ) < 0$ and $B_{5} - q_{21} B_{6} < 0$; (3) $(B_{3} - C_{21} ) - q_{21} (B_{4} - C_{21} ) > 0$, $B_{5} - q_{21} B_{6} < 0$ and ${(}B_{1} - C_{1} - C_{11} + C_{21} {)} - p_{21} {(}B_{1} - C_{1} - C_{11} {) < 0}$; (3) $B_{5} - q_{21} B_{6} < 0$,$(B_{3} - C_{21} ) - q_{21} (B_{4} - C_{21} ) < 0$ and ${(}B_{1} - C_{1} - C_{11} + C_{21} {)} - p_{21} {(}B_{1} - C_{1} - C_{11} {) > 0}$; (4)${(}B_{1} - C_{1} - C_{11} + C_{21} {)} - p_{21} {(}B_{1} - C_{1} - C_{11} {) > 0}$, $(B_{3} - C_{21} ) - q_{21} (B_{4} - C_{21} ) > 0$ and $B_{5} - q_{21} B_{6} < 0$; (5)${(}B_{1} - C_{1} - C_{11} + C_{21} {)} - p_{21} {(}B_{1} - C_{1} - C_{11} {) > 0}$, $B_{5} - q_{21} B_{6} > 0$ and $(B_{3} - C_{21} ) - q_{21} (B_{4} - C_{21} ) < 0$; (6) ${(}B_{1} - C_{1} - C_{11} + C_{21} {)} - p_{21} {(}B_{1} - C_{1} - C_{11} {) < 0}$, $(B_{3} - C_{21} ) - q_{21} (B_{4} - C_{21} ) > 0$ and $B_{5} - q_{21} B_{6} > 0$; (7) $(B_{3} - C_{21} ) - q_{21} (B_{4} - C_{21} ) < 0$, $B_{5} - q_{21} B_{6} > 0$ and ${(}B_{1} - C_{1} - C_{11} + C_{21} {)} - p_{21} {(}B_{1} - C_{1} - C_{11} {) < 0}$; (8) $B_{5} - q_{21} B_{6} > 0$, $(B_{3} - C_{21} ) - q_{21} (B_{4} - C_{21} ) > 0$ and ${(}B_{1} - C_{1} - C_{11} + C_{21} {)} - p_{21} {(}B_{1} - C_{1} - C_{11} {) > 0}$. The results of analysis are shown in Table
  **Table 4:** Evolutionary stability analysis of the model($B_{5} - B_{3} + C_{21} + q_{21} (B_{4} - C_{21} ) - q_{21} B_{6} \ne 0$, $(1 - p_{21} )(B_{2} + C_{11} - B_{1} ) = 0$).
  Case	Basis of Judgment	(0,0)	(0,1)	(1,0)	(1,1)
  Case 1	$\det J$	+	−	−	+
  	$trJ$	−	Uncertainty	Uncertainty	+
  	Stability	ESS	Saddle point	Saddle point	Unstable point
  Case 2	$\det J$	+	+	−	−
  	$trJ$	−	+	Uncertainty	Uncertainty
  	Stability	ESS	Unstable point	Saddle point	Saddle point
  Case 3	$\det J$	−	+	+	−
  	$trJ$	Uncertainty	+	−	Uncertainty
  	Stability	Saddle point	Unstable point	ESS	Saddle point
  Case 4	$\det J$	−	+	−	+
  	$trJ$	Uncertainty	+	Uncertainty	−
  	Stability	Saddle point	Unstable point	Saddle point	ESS
  Case 5	$\det J$	+	−	+	−
  	$trJ$	+	Uncertainty	−	Uncertainty
  	Stability	Unstable point	Saddle point	ESS	Saddle point
  Case 6	$\det J$	−	+	+	−
  	$trJ$	Uncertainty	−	+	Uncertainty
  	Stability	Saddle point	ESS	Unstable point	Saddle point
  Case 7	$\det J$	−	+	−	+
  	$trJ$	Uncertainty	−	Uncertainty	+
  	Stability	Saddle point	ESS	Saddle point	Unstable point
  Case 8	$\det J$	+	−	−	+
  	$trJ$	+	Uncertainty	Uncertainty	−
  	Stability	Unstable point	Saddle point	Saddle point	ESS
  4.

## Appendix A.6.2

(IV) When $B_{5} - B_{3} + C_{21} + q_{21} (B_{4} - C_{21} ) - q_{21} B_{6} \ne 0$ and $(1 - p_{21} )(B_{2} + C_{11} - B_{1} ) \ne 0$, the five equilibrium points of the evolutionary game model are $E_{1} (0,0)$, $E_{2} (0,1)$, $E_{5} (\frac{{B_{5} - q_{21} B_{6} }}{{B_{5} - B_{3} + C_{21} + q_{21} (B_{4} - C_{21} ) - q_{21} B_{6} }},\frac{{B_{2} - C_{1} + C_{21} - p_{21} (B_{2} - C_{1} )}}{{(1 - p_{21} )(B_{2} + C_{11} - B_{1} )}})$, $E_{3} (1,0)$ and $E_{4} (1,1)$, respectively. Several situations need to be discussed.
(1) When $\frac{{B_{5} - q_{21} B_{6} }}{{B_{5} - B_{3} + C_{21} + q_{21} (B_{4} - C_{21} ) - q_{21} B_{6} }} < 0,\frac{{B_{2} - C_{1} + C_{21} - p_{21} (B_{2} - C_{1} )}}{{(1 - p_{21} )(B_{2} + C_{11} - B_{1} )}} < 0$, the following four cases are discussed:
(i) When $0 < B_{2} - C_{1} + C_{21} - p_{21} (B_{2} - C_{1} ) < {(}B_{1} - C_{1} - C_{11} + C_{21} {)} - p_{21} {(}B_{1} - C_{1} - C_{11} {)}$, $0 < B_{5} - q_{21} B_{6} < B_{3} - C_{21} - q_{21} (B_{4} - C_{21} )$, $E_{4} (1,1)$ is the stable point, $E_{2} (0,1)$, $E_{3} (1,0)$ and $E_{5} (x^{ * } ,y^{ * } )$ are the saddle points, and $E_{1} (0,0)$ is an unstable point.
(ii) When ${(}B_{1} - C_{1} - C_{11} + C_{21} {)} - p_{21} {(}B_{1} - C_{1} - C_{11} {)} < B_{2} - C_{1} + C_{21} - p_{21} (B_{2} - C_{1} ) < 0$, $0 < B_{5} - q_{21} B_{6} < B_{3} - C_{21} - q_{21} (B_{4} - C_{21} )$, $E_{2} (0,1)$ is the stable point, $E_{1} (0,0)$ and $E_{4} (1,1)$ are the saddle points, $E_{3} (1,0)$ is an unstable point , and $E_{5} (x^{ * } ,y^{ * } )$ is the center point.
(iii) When ${(}B_{1} - C_{1} - C_{11} + C_{21} {)} - p_{21} {(}B_{1} - C_{1} - C_{11} {)} < B_{2} - C_{1} + C_{21} - p_{21} (B_{2} - C_{1} ) < 0$, $B_{3} - C_{21} - q_{21} (B_{4} - C_{21} ) < B_{5} - q_{21} B_{6} < 0$, $E_{1} (0,0)$ is the stable point, $E_{2} (0,1)$, $E_{3} (1,0)$ and $E_{5} (x^{ * } ,y^{ * } )$ are the saddle points, and $E_{4} (1,1)$ is an unstable point.
(iv) When $B_{3} - C_{21} - q_{21} (B_{4} - C_{21} ) < B_{5} - q_{21} B_{6} < 0$, $0 < B_{2} - C_{1} + C_{21} - p_{21} (B_{2} - C_{1} ) < {(}B_{1} - C_{1} - C_{11} + C_{21} {)} - p_{21} {(}B_{1} - C_{1} - C_{11} {)}$, $E_{3} (1,0)$ is the stable point, $E_{1} (0,0)$ and $E_{4} (1,1)$ are the saddle points, $E_{2} (0,1)$ is an unstable point, and $E_{5} (x^{ * } ,y^{ * } )$ is the center point.
(2) When $0 < \frac{{B_{5} - q_{21} B_{6} }}{{B_{5} - B_{3} + C_{21} + q_{21} (B_{4} - C_{21} ) - q_{21} B_{6} }} < 1,\frac{{B_{2} - C_{1} + C_{21} - p_{21} (B_{2} - C_{1} )}}{{(1 - p_{21} )(B_{2} + C_{11} - B_{1} )}} < 0$, the following four cases are discussed:
(i) When $0 < B_{2} - C_{1} + C_{21} - p_{21} (B_{2} - C_{1} ) < {(}B_{1} - C_{1} - C_{11} + C_{21} {)} - p_{21} {(}B_{1} - C_{1} - C_{11} {)}$, $B_{3} - C_{21} - q_{21} (B_{4} - C_{21} ) < 0 < B_{5} - q_{21} B_{6}$, $E_{3} (1,0)$ is the stable point, $E_{2} (0,1)$, $E_{4} (1,1)$ and $E_{5} (x^{ * } ,y^{ * } )$ are the saddle points, and $E_{1} (0,0)$ is an unstable point.
(ii) When ${(}B_{1} - C_{1} - C_{11} + C_{21} {)} - p_{21} {(}B_{1} - C_{1} - C_{11} {)} < B_{2} - C_{1} + C_{21} - p_{21} (B_{2} - C_{1} ) < 0$, $B_{3} - C_{21} - q_{21} (B_{4} - C_{21} ) < 0 < B_{5} - q_{21} B_{6}$, $E_{2} (0,1)$ is the stable point, $E_{1} (0,0)$ and $E_{3} (1,0)$ are the saddle points, $E_{4} (1,1)$ is an unstable point , and $E_{5} (x^{ * } ,y^{ * } )$ is the center point.
(iii) When ${(}B_{1} - C_{1} - C_{11} + C_{21} {)} - p_{21} {(}B_{1} - C_{1} - C_{11} {)} < B_{2} - C_{1} + C_{21} - p_{21} (B_{2} - C_{1} ) < 0$, $B_{5} - q_{21} B_{6} < 0 < B_{3} - C_{21} - q_{21} (B_{4} - C_{21} )$, $E_{1} (0,0)$ is the stable point, $E_{2} (0,1)$, $E_{4} (1,1)$ and $E_{5} (x^{ * } ,y^{ * } )$ are the saddle points, and $E_{3} (1,0)$ is an unstable point.
(iv) When $B_{5} - q_{21} B_{6} < 0 < B_{3} - C_{21} - q_{21} (B_{4} - C_{21} )$, $0 < B_{2} - C_{1} + C_{21} - p_{21} (B_{2} - C_{1} ) < {(}B_{1} - C_{1} - C_{11} + C_{21} {)} - p_{21} {(}B_{1} - C_{1} - C_{11} {)}$, $E_{4} (1,1)$ is the stable point, $E_{1} (0,0)$ and $E_{3} (1,0)$ are the saddle points, $E_{2} (0,1)$ is an unstable point, and $E_{5} (x^{ * } ,y^{ * } )$ is the center point.
(3) When $\frac{{B_{5} - q_{21} B_{6} }}{{B_{5} - B_{3} + C_{21} + q_{21} (B_{4} - C_{21} ) - q_{21} B_{6} }} > 1,\frac{{B_{2} - C_{1} + C_{21} - p_{21} (B_{2} - C_{1} )}}{{(1 - p_{21} )(B_{2} + C_{11} - B_{1} )}} < 0$, the following four cases are discussed:
(i) When $0 < B_{2} - C_{1} + C_{21} - p_{21} (B_{2} - C_{1} ) < {(}B_{1} - C_{1} - C_{11} + C_{21} {)} - p_{21} {(}B_{1} - C_{1} - C_{11} {)}$, $0 < B_{3} - C_{21} - q_{21} (B_{4} - C_{21} ) < B_{5} - q_{21} B_{6}$, $E_{4} (1,1)$ is the stable point, $E_{2} (0,1)$ and $E_{3} (1,0)$ are the saddle points, $E_{1} (0,0)$ is an unstable point, and $E_{5} (x^{ * } ,y^{ * } )$ is the center point.
(ii) When ${(}B_{1} - C_{1} - C_{11} + C_{21} {)} - p_{21} {(}B_{1} - C_{1} - C_{11} {)} < B_{2} - C_{1} + C_{21} - p_{21} (B_{2} - C_{1} ) < 0$, $0 < B_{3} - C_{21} - q_{21} (B_{4} - C_{21} ) < B_{5} - q_{21} B_{6}$, $E_{2} (0,1)$ is the stable point, $E_{1} (0,0)$, $E_{4} (1,1)$ and $E_{5} (x^{ * } ,y^{ * } )$ are the saddle points, and $E_{3} (1,0)$ is an unstable point.
(iii) When $0 < B_{2} - C_{1} + C_{21} - p_{21} (B_{2} - C_{1} ) < {(}B_{1} - C_{1} - C_{11} + C_{21} {)} - p_{21} {(}B_{1} - C_{1} - C_{11} {)}$, $B_{5} - q_{21} B_{6} < B_{3} - C_{21} - q_{21} (B_{4} - C_{21} ) < 0$, $E_{3} (1,0)$ is the stable point, $E_{1} (0,0)$, $E_{4} (1,1)$ and $E_{5} (x^{ * } ,y^{ * } )$ are the saddle points, and $E_{2} (0,1)$ is an unstable point.
(iv) When $B_{5} - q_{21} B_{6} < B_{3} - C_{21} - q_{21} (B_{4} - C_{21} ) < 0$, ${(}B_{1} - C_{1} - C_{11} + C_{21} {)} - p_{21} {(}B_{1} - C_{1} - C_{11} {)} < B_{2} - C_{1} + C_{21} - p_{21} (B_{2} - C_{1} ) < 0$, $E_{1} (0,0)$ is the stable point, $E_{2} (0,1)$ and $E_{3} (1,0)$ are the saddle points, $E_{4} (1,1)$ is an unstable point, and $E_{5} (x^{ * } ,y^{ * } )$ is the center point.
(4) When $\frac{{B_{5} - q_{21} B_{6} }}{{B_{5} - B_{3} + C_{21} + q_{21} (B_{4} - C_{21} ) - q_{21} B_{6} }} < 0,0 < \frac{{B_{2} - C_{1} + C_{21} - p_{21} (B_{2} - C_{1} )}}{{(1 - p_{21} )(B_{2} + C_{11} - B_{1} )}} < 1$, the following four cases are discussed:
(i) When ${(}B_{1} - C_{1} - C_{11} + C_{21} {)} - p_{21} {(}B_{1} - C_{1} - C_{11} {)} < 0 < B_{2} - C_{1} + C_{21} - p_{21} (B_{2} - C_{1} )$, $0 < B_{5} - q_{21} B_{6} < B_{3} - C_{21} - q_{21} (B_{4} - C_{21} )$, $E_{2} (0,1)$ is the stable point, $E_{3} (1,0)$, $E_{4} (1,1)$ and $E_{5} (x^{ * } ,y^{ * } )$ are the saddle points, and $E_{1} (0,0)$ is an unstable point.
(ii) When $B_{2} - C_{1} + C_{21} - p_{21} (B_{2} - C_{1} ) < 0 < {(}B_{1} - C_{1} - C_{11} + C_{21} {)} - p_{21} {(}B_{1} - C_{1} - C_{11} {)}$, $0 < B_{5} - q_{21} B_{6} < B_{3} - C_{21} - q_{21} (B_{4} - C_{21} )$, $E_{4} (1,1)$ is the stable point, $E_{1} (0,0)$ and $E_{2} (0,1)$ are the saddle points, $E_{3} (1,0)$ is an unstable point, and $E_{5} (x^{ * } ,y^{ * } )$ is the center point.
(iii) When ${(}B_{1} - C_{1} - C_{11} + C_{21} {)} - p_{21} {(}B_{1} - C_{1} - C_{11} {)} < 0 < B_{2} - C_{1} + C_{21} - p_{21} (B_{2} - C_{1} )$, $B_{3} - C_{21} - q_{21} (B_{4} - C_{21} ) < B_{5} - q_{21} B_{6} < 0$, $E_{3} (1,0)$ is the stable point, $E_{1} (0,0)$ and $E_{2} (0,1)$ are the saddle points, $E_{4} (1,1)$ is an unstable point, and $E_{5} (x^{ * } ,y^{ * } )$ is the center point.
(iv) When $B_{3} - C_{21} - q_{21} (B_{4} - C_{21} ) < B_{5} - q_{21} B_{6} < 0$, $B_{2} - C_{1} + C_{21} - p_{21} (B_{2} - C_{1} ) < 0 < {(}B_{1} - C_{1} - C_{11} + C_{21} {)} - p_{21} {(}B_{1} - C_{1} - C_{11} {)}$, $E_{1} (0,0)$ is the stable point, $E_{3} (1,0)$, $E_{4} (1,1)$ and $E_{5} (x^{ * } ,y^{ * } )$ are the saddle points, and $E_{2} (0,1)$ is an unstable point.
(5) When $\frac{{B_{5} - q_{21} B_{6} }}{{B_{5} - B_{3} + C_{21} + q_{21} (B_{4} - C_{21} ) - q_{21} B_{6} }} < 0,\frac{{B_{2} - C_{1} + C_{21} - p_{21} (B_{2} - C_{1} )}}{{(1 - p_{21} )(B_{2} + C_{11} - B_{1} )}} > 1$, the following four cases are discussed:
(i) When $0 < {(}B_{1} - C_{1} - C_{11} + C_{21} {)} - p_{21} {(}B_{1} - C_{1} - C_{11} {)} < B_{2} - C_{1} + C_{21} - p_{21} (B_{2} - C_{1} )$, $0 < B_{5} - q_{21} B_{6} < B_{3} - C_{21} - q_{21} (B_{4} - C_{21} )$, $E_{4} (1,1)$ is the stable point, $E_{2} (0,1)$ and $E_{3} (1,0)$ are the saddle points, $E_{1} (0,0)$ is an unstable point, and $E_{5} (x^{ * } ,y^{ * } )$ is the center point.
(ii) When $B_{2} - C_{1} + C_{21} - p_{21} (B_{2} - C_{1} ) < {(}B_{1} - C_{1} - C_{11} + C_{21} {)} - p_{21} {(}B_{1} - C_{1} - C_{11} {)} < 0$, $0 < B_{5} - q_{21} B_{6} < B_{3} - C_{21} - q_{21} (B_{4} - C_{21} )$, $E_{2} (0,1)$ is the stable point, $E_{1} (0,0)$, $E_{4} (1,1)$ and $E_{5} (x^{ * } ,y^{ * } )$ are the saddle points, and $E_{3} (1,0)$ is an unstable point.
(iii) When $B_{2} - C_{1} + C_{21} - p_{21} (B_{2} - C_{1} ) < {(}B_{1} - C_{1} - C_{11} + C_{21} {)} - p_{21} {(}B_{1} - C_{1} - C_{11} {)} < 0$, $B_{3} - C_{21} - q_{21} (B_{4} - C_{21} ) < B_{5} - q_{21} B_{6} < 0$ 时, $E_{1} (0,0)$ is the stable point, $E_{2} (0,1)$ and $E_{3} (1,0)$ are the saddle points, $E_{4} (1,1)$ is an unstable point, and $E_{5} (x^{ * } ,y^{ * } )$ is the center point.
(iv) When $B_{3} - C_{21} - q_{21} (B_{4} - C_{21} ) < B_{5} - q_{21} B_{6} < 0$, $0 < {(}B_{1} - C_{1} - C_{11} + C_{21} {)} - p_{21} {(}B_{1} - C_{1} - C_{11} {)} < B_{2} - C_{1} + C_{21} - p_{21} (B_{2} - C_{1} )$, $E_{3} (1,0)$ is the stable point, $E_{1} (0,0)$, $E_{4} (1,1)$ and $E_{5} (x^{ * } ,y^{ * } )$ are the saddle points, $E_{2} (0,1)$ is an unstable point.
(6) When $0 < \frac{{B_{5} - q_{21} B_{6} }}{{B_{5} - B_{3} + C_{21} + q_{21} (B_{4} - C_{21} ) - q_{21} B_{6} }} < 1$,$0 < \frac{{B_{2} - C_{1} + C_{21} - p_{21} (B_{2} - C_{1} )}}{{(1 - p_{21} )(B_{2} + C_{11} - B_{1} )}} < 1$, the following four cases are discussed:
(i) When $B_{2} - C_{1} + C_{21} - p_{21} (B_{2} - C_{1} ) < 0 < {(}B_{1} - C_{1} - C_{11} + C_{21} {)} - p_{21} {(}B_{1} - C_{1} - C_{11} {)}$, $B_{3} - C_{21} - q_{21} (B_{4} - C_{21} ) < 0 < B_{5} - q_{21} B_{6}$, $E_{1} (0,0)$ and $E_{4} (1,1)$ are the stable point, $E_{5} (x^{ * } ,y^{ * } )$ are the saddle points, and $E_{2} (0,1)$ and $E_{3} (1,0)$ are an unstable point.
(ii) When ${(}B_{1} - C_{1} - C_{11} + C_{21} {)} - p_{21} {(}B_{1} - C_{1} - C_{11} {)} < 0 < B_{2} - C_{1} + C_{21} - p_{21} (B_{2} - C_{1} )$, $B_{3} - C_{21} - q_{21} (B_{4} - C_{21} ) < 0 < B_{5} - q_{21} B_{6}$ 时, $E_{1} (0,0)$, $E_{2} (0,1)$, $E_{3} (1,0)$ and $E_{4} (1,1)$ are the saddle points, is an unstable point, and $E_{5} (x^{ * } ,y^{ * } )$ is the center point.
(iii) When $B_{2} - C_{1} + C_{21} - p_{21} (B_{2} - C_{1} ) < 0 < {(}B_{1} - C_{1} - C_{11} + C_{21} {)} - p_{21} {(}B_{1} - C_{1} - C_{11} {)}$, $B_{5} - q_{21} B_{6} < 0 < B_{3} - C_{21} - q_{21} (B_{4} - C_{21} )$, $E_{2} (0,1)$ and $E_{3} (1,0)$ are the stable point, $E_{5} (x^{ * } ,y^{ * } )$ are the saddle points, and $E_{1} (0,0)$ and $E_{4} (1,1)$ are an unstable point.
(iv) When $B_{5} - q_{21} B_{6} < 0 < B_{3} - C_{21} - q_{21} (B_{4} - C_{21} )$,${(}B_{1} - C_{1} - C_{11} + C_{21} {)} - p_{21} {(}B_{1} - C_{1} - C_{11} {)} < 0 < B_{2} - C_{1} + C_{21} - p_{21} (B_{2} - C_{1} )$, 时, $E_{1} (0,0)$, $E_{2} (0,1)$, $E_{3} (1,0)$ and $E_{4} (1,1)$ are the saddle points, is an unstable point, and $E_{5} (x^{ * } ,y^{ * } )$ is the center point.
(7) When $0 < \frac{{B_{5} - q_{21} B_{6} }}{{B_{5} - B_{3} + C_{21} + q_{21} (B_{4} - C_{21} ) - q_{21} B_{6} }} < 1,\frac{{B_{2} - C_{1} + C_{21} - p_{21} (B_{2} - C_{1} )}}{{(1 - p_{21} )(B_{2} + C_{11} - B_{1} )}} > 1$, the following four cases are discussed:
(i) When $0 < {(}B_{1} - C_{1} - C_{11} + C_{21} {)} - p_{21} {(}B_{1} - C_{1} - C_{11} {)} < B_{2} - C_{1} + C_{21} - p_{21} (B_{2} - C_{1} )$, $B_{3} - C_{21} - q_{21} (B_{4} - C_{21} ) < 0 < B_{5} - q_{21} B_{6}$, $E_{3} (1,0)$ is the stable point, $E_{2} (0,1)$ and $E_{4} (1,1)$ are the saddle points, $E_{1} (0,0)$ is an unstable point, and $E_{5} (x^{ * } ,y^{ * } )$ is the center point.
(ii) When $B_{2} - C_{1} + C_{21} - p_{21} (B_{2} - C_{1} ) < {(}B_{1} - C_{1} - C_{11} + C_{21} {)} - p_{21} {(}B_{1} - C_{1} - C_{11} {)} < 0$, $B_{3} - C_{21} - q_{21} (B_{4} - C_{21} ) < 0 < B_{5} - q_{21} B_{6}$, $E_{2} (0,1)$ is the stable point, $E_{1} (0,0)$, $E_{3} (1,0)$ and $E_{5} (x^{ * } ,y^{ * } )$ are the saddle points, $E_{4} (1,1)$ is an unstable point, and is the center point.
(iii) When $0 < {(}B_{1} - C_{1} - C_{11} + C_{21} {)} - p_{21} {(}B_{1} - C_{1} - C_{11} {)} < B_{2} - C_{1} + C_{21} - p_{21} (B_{2} - C_{1} )$, $B_{5} - q_{21} B_{6} < 0 < B_{3} - C_{21} - q_{21} (B_{4} - C_{21} )$, $E_{3} (1,0)$ is the stable point, $E_{1} (0,0)$, $E_{4} (1,1)$ and $E_{5} (x^{ * } ,y^{ * } )$ are the saddle points, $E_{2} (0,1)$ is an unstable point, and is the center point.
(iv) When $B_{5} - q_{21} B_{6} < 0 < B_{3} - C_{21} - q_{21} (B_{4} - C_{21} )$, $B_{2} - C_{1} + C_{21} - p_{21} (B_{2} - C_{1} ) < {(}B_{1} - C_{1} - C_{11} + C_{21} {)} - p_{21} {(}B_{1} - C_{1} - C_{11} {)} < 0$, $E_{1} (0,0)$ is the stable point, $E_{2} (0,1)$ and $E_{4} (1,1)$ are the saddle points, $E_{3} (1,0)$ is an unstable point, and $E_{5} (x^{ * } ,y^{ * } )$ is the center point.
(8) When $\frac{{B_{5} - q_{21} B_{6} }}{{B_{5} - B_{3} + C_{21} + q_{21} (B_{4} - C_{21} ) - q_{21} B_{6} }} > 1,0 < \frac{{B_{2} - C_{1} + C_{21} - p_{21} (B_{2} - C_{1} )}}{{(1 - p_{21} )(B_{2} + C_{11} - B_{1} )}} < 1$, the following four cases are discussed:
(i) When ${(}B_{1} - C_{1} - C_{11} + C_{21} {)} - p_{21} {(}B_{1} - C_{1} - C_{11} {)} < 0 < B_{2} - C_{1} + C_{21} - p_{21} (B_{2} - C_{1} )$, $0 < B_{3} - C_{21} - q_{21} (B_{4} - C_{21} ) < B_{5} - q_{21} B_{6}$, $E_{2} (0,1)$ is the stable point, $E_{3} (1,0)$ and $E_{4} (1,1)$ are the saddle points, $E_{1} (0,0)$ is an unstable point, and $E_{5} (x^{ * } ,y^{ * } )$ is the center point.
(ii) When $B_{2} - C_{1} + C_{21} - p_{21} (B_{2} - C_{1} ) < 0 < {(}B_{1} - C_{1} - C_{11} + C_{21} {)} - p_{21} {(}B_{1} - C_{1} - C_{11} {)}$, $0 < B_{3} - C_{21} - q_{21} (B_{4} - C_{21} ) < B_{5} - q_{21} B_{6}$, $E_{4} (1,1)$ is the stable point, $E_{1} (0,0)$, $E_{2} (0,1)$ and $E_{5} (x^{ * } ,y^{ * } )$ are the saddle points, $E_{3} (1,0)$ is an unstable point.
(iii) When ${(}B_{1} - C_{1} - C_{11} + C_{21} {)} - p_{21} {(}B_{1} - C_{1} - C_{11} {)} < 0 < B_{2} - C_{1} + C_{21} - p_{21} (B_{2} - C_{1} )$, $B_{5} - q_{21} B_{6} < B_{3} - C_{21} - q_{21} (B_{4} - C_{21} ) < 0$, $E_{1} (0,0)$ is the stable point, $E_{3} (1,0)$ and $E_{4} (1,1)$ are the saddle points, $E_{2} (0,1)$ is an unstable point, and $E_{5} (x^{ * } ,y^{ * } )$ is the center point.
(iv) When $B_{5} - q_{21} B_{6} < B_{3} - C_{21} - q_{21} (B_{4} - C_{21} ) < 0$, $B_{2} - C_{1} + C_{21} - p_{21} (B_{2} - C_{1} ) < 0 < {(}B_{1} - C_{1} - C_{11} + C_{21} {)} - p_{21} {(}B_{1} - C_{1} - C_{11} {)}$, $E_{3} (1,0)$ is the stable point, $E_{1} (0,0)$, $E_{2} (0,1)$ and $E_{5} (x^{ * } ,y^{ * } )$ are the saddle points, $E_{4} (1,1)$ is an unstable point.
(9) When $\frac{{B_{5} - q_{21} B_{6} }}{{B_{5} - B_{3} + C_{21} + q_{21} (B_{4} - C_{21} ) - q_{21} B_{6} }} > 1,\frac{{B_{2} - C_{1} + C_{21} - p_{21} (B_{2} - C_{1} )}}{{(1 - p_{21} )(B_{2} + C_{11} - B_{1} )}} > 1$, the following four cases are discussed:
(i) When $0 < {(}B_{1} - C_{1} - C_{11} + C_{21} {)} - p_{21} {(}B_{1} - C_{1} - C_{11} {)} < B_{2} - C_{1} + C_{21} - p_{21} (B_{2} - C_{1} )$, $0 < B_{3} - C_{21} - q_{21} (B_{4} - C_{21} ) < B_{5} - q_{21} B_{6}$, $E_{4} (1,1)$ is the stable point, $E_{2} (0,1)$, $E_{3} (1,0)$ and $E_{5} (x^{ * } ,y^{ * } )$ are the saddle points, $E_{1} (0,0)$ is an unstable point.
(ii) When $B_{2} - C_{1} + C_{21} - p_{21} (B_{2} - C_{1} ) < {(}B_{1} - C_{1} - C_{11} + C_{21} {)} - p_{21} {(}B_{1} - C_{1} - C_{11} {)} < 0$, $0 < B_{3} - C_{21} - q_{21} (B_{4} - C_{21} ) < B_{5} - q_{21} B_{6}$, $E_{2} (0,1)$ is the stable point, $E_{1} (0,0)$ and $E_{4} (1,1)$ are the saddle points, $E_{3} (1,0)$ is an unstable point, and $E_{5} (x^{ * } ,y^{ * } )$ is the center point.
(iii) When $0 < {(}B_{1} - C_{1} - C_{11} + C_{21} {)} - p_{21} {(}B_{1} - C_{1} - C_{11} {)} < B_{2} - C_{1} + C_{21} - p_{21} (B_{2} - C_{1} )$, $B_{5} - q_{21} B_{6} < B_{3} - C_{21} - q_{21} (B_{4} - C_{21} ) < 0$, $E_{1} (0,0)$ is the stable point, $E_{2} (0,1)$, $E_{3} (1,0)$ and $E_{5} (x^{ * } ,y^{ * } )$ are the saddle points, $E_{4} (1,1)$ is an unstable point.
(iv) When $B_{5} - q_{21} B_{6} < B_{3} - C_{21} - q_{21} (B_{4} - C_{21} ) < 0$, $B_{2} - C_{1} + C_{21} - p_{21} (B_{2} - C_{1} ) < {(}B_{1} - C_{1} - C_{11} + C_{21} {)} - p_{21} {(}B_{1} - C_{1} - C_{11} {)} < 0$, $E_{3} (1,0)$ is the stable point, $E_{1} (0,0)$ and $E_{4} (1,1)$ are the saddle points, $E_{2} (0,1)$ is an unstable point, and $E_{5} (x^{ * } ,y^{ * } )$ is the center point.

## References

[1] Hu X, Naim K, Jia S (2021). Disaster policy and emergency management reforms in China: From Wenchuan earthquake to Jiuzhaigou earthquake. Int. J. Disaster Risk Reduct..

[2] Zhao T, Wang J, Sun L (2023). Crowd dynamic-based model on the city-wide emergency transfer under catastrophic earthquakes. Int. J. Disaster Risk Reduct..

[3] Gabrick EC, Protachevicz PR, Batista AM (2022). Effect of two vaccine doses in the SEIR epidemic model using a stochastic cellular automaton. Phys. A Stat. Mech. Appl..

[4] Andrejko KL, Myers JF, Openshaw J (2023). Receipt of COVID-19 and seasonal influenza vaccines in California (USA) during the 2021–2022 influenza season. Vaccine.

[5] Avinash Sharma BM, Hui DS (2023). Global mass gathering events and deaths due to crowd surge, stampedes, crush and physical injuries – Lessons from the Seoul Halloween and other disasters. Travel Med. Infect. Dis..

[6] Huo F, Li Y, Li C (2022). An extended model describing pedestrian evacuation considering pedestrian crowding and stampede behavior. Phys. A Stat. Mech. Appl..

[7] Shi Y, Zhang G, Lu D (2021). Intervention optimization for crowd emotional contagion. Inf. Sci..

[8] XU M, XIE X, LV P, *et al.* Crowd Behavior Simulation with Emotional Contagion in Unexpected Multi-hazard Situations.*IEEE transactions on systems,man and cybernetics System*s,**51**(3), 1567–1581. 10.1109/tsmc.2019.2899047 (2021).

[9] Sun L, Rao Y, Wu L (2023). Fighting false information from propagation process: A survey. ACM Comput. Surv..

[10] Kermack WO, McKendrick AGA (1927). A contribution to the mathematical theory of epidemics. Proc. R. Soc. A Math. Phys. Eng. Sci..

[11] Hezam IM, Almshnanah A, Mubarak AA (2023). COVID-19 and rumors: A dynamic nested optimal control model. Pattern Recognit..

[12] Jiang J, Gong S, He B (2016). Dynamical behavior of a rumor transmission model with Holling-type II functional response in emergency event. Phys. A Stat. Mech. Appl..

[13] Hu Y, Pan Q, Hou W (2018). Rumor spreading model considering the proportion of wisemen in the crowd. Phys. A-Stat. Mech. Appl..

[14] Hu Y, Pan Q, Hou W (2018). Rumor spreading model with the different attitudes towards rumors. Phys. A-Stat. Mech. Appl..

[15] Tian Y, Ding X (2019). Rumor spreading model with considering debunking behavior in emergencies. Appl. Math. Comput..

[16] Yi J, Liu P, Wang Z (2021). Research on twin-SIR rumor spreading model in online social network. J. Intell. Fuzzy Syst..

[17] Cheng Y, Huo L, Zhao L (2021). Dynamical behaviors and control measures of rumor-spreading model in consideration of the infected media and time delay. Inf. Sci..

[18] Hu J, Zhu L (2021). Turing pattern analysis of a reaction-diffusion rumor propagation system with time delay in both network and non-network environments. Chaos Solitons Fractals.

[19] Zhang GJ, Lu DJ, Liu H (2021). IoT-based positive emotional contagion for crowd evacuation. IEEE Internet Things J..

[20] van Haeringen ES, Gerritsen C, Hindriks KV (2023). Emotion contagion in agent-based simulations of crowds: A systematic review. Auton. Agents Multi-Agent Syst..

[21] Xu T, Shi D, Chen J (2019). Dynamics of emotional contagion in dense pedestrian crowds. Phys. Lett. A.

[22] Liu Z, Liu T, Ma M (2018). A perception-based emotion contagion model in crowd emergent evacuation simulation. Comput. Anim. Virtual Worlds.

[23] Li Wenqian, Z. L. Dynamics analysis of panic emotion propagation model considering delay effect. In *Proceedings of the**The First Graduate Forum of the CSAA and The Seventh International Academic Conference for Graduates, Nanjing**: **NUAA, F*. (2019).

[24] Xia Y, Jiang H, Yu Z (2023). Dynamic analysis and optimal control of a reaction-diffusion rumor propagation model in multi-lingual environments. J. Math. Anal. Appl..

[25] Chen L, Jiang H, Li L (2020). Dynamical behaviors and optimal control of rumor propagation model with saturation incidence on heterogeneous networks. Chaos Solitons Fractals Appl. Sci. Eng. Interdiscip. J. Nonlinear Sci..

[26] Cheng Y, Huo L, Zhao L (2022). Stability analysis and optimal control of rumor spreading model under media coverage considering time delay and pulse vaccination. Chaos Solitons Fractals.

[27] Wang X, Zhang L, Lin Y (2016). Computational models and optimal control strategies for emotion contagion in the human population in emergencies. Knowl. -Based Sys..

[28] Zhang M, Kong Z (2022). A tripartite evolutionary game model of emergency supplies joint reserve among the government, enterprise and society. Comput. Ind. Eng..

[29] Shao Q, Yuan J (2022). Study on the disposal strategy of civil aviation passenger collective events based on evolutionary game theory. Phys. A-Stat. Mech. Appl..

[30] Li B, Li H, Sun Q (2020). Evolutionary game analysis of online collective behaviour with the introduction of the degree of psychological identity. Behav. Inf. Technol..

[31] Li B, Li H, Sun Q (2020). Evolutionary game analysis between businesses and consumers under the background of Internet rumors. Concurr. Comput. –Pract. Exp..

[32] Wang L, Schuetz CG, Cai D (2021). Choosing response strategies in social media crisis communication: An evolutionary game theory perspective. Inf. Manag..

[33] Shi W, Wang H, Chen C (2021). Evolutionary game analysis of decision-making dynamics of local governments and residents during wildfires. Int. J. Disaster Risk Reduct..

[34] Kabir KMA, Chowdhury A, Tanimoto J (2021). An evolutionary game modeling to assess the effect of border enforcement measures and socio-economic cost: Export-importation epidemic dynamics. Chaos Solitons Fractals.

[35] Wang T, Huang K, Cheng Y (2015). Understanding herding based on a co-evolutionary model for strategy and game structure. Chaos Solitons Fractals.

[36] Shen S, Huang L, Fan E (2016). Trust dynamics in WSNs: An evolutionary game-theoretic approach. J. Sens..

[37] De la Sen M, Ibeas A, Alonso-Quesada S (2012). On vaccination controls for the SEIR epidemic model. Commun. Nonlinear Sci. Numer. Simul..

[38] Holling CS (1965). The Functional response of predators to prey density ^id its role in mimicry and population regulation. Memoirs Entomol. Soc. Canada.

[39] Lee WK, Fan W, Miller M (2022). Toward cost-sensitive modeling for intrusion detection and response. J. Comput. Secur..

[40] Borkovsky RN, Doraszelski U, Kryukov Y (2015). A user’ s guide to solving dynamic stochastic games using the homotopy method. Oper. Res..

[41] van den Driessche P, Watmough J (2002). Reproduction numbers and sub- threshold endemic equilibria for compaxtmental models of disease transmission. Math. Biosci..

[42] LaSalle JP (1976). The Stability of Dynamical Systems.

[43] Hurwitz A (1895). Ueber die bedingungen, unter welchen eine gleichung nur wurzeln mit negativen reellen theilen besitzt [J]. Math. Ann..

